# Discovery of chirally dependent protein modifications by D- and L-2-hydroxyglutarates

**DOI:** 10.1038/s41557-026-02093-x

**Published:** 2026-03-17

**Authors:** Zheng Zhang, Yi-Kai Liu, Zhuojun Luo, Meng-Ju Wu, Claudia N. Evans, Zihan Qu, Fanglei Xue, Zhijian Wang, Lia Stanciu, Zhong-Yin Zhang, Elizabeth I. Parkinson, Nabeel Bardeesy, W. Andy Tao

**Affiliations:** 1 Department of Biochemistry, Purdue University, West Lafayette, IN, USA; 2 Cancer Center, Massachusetts General Hospital, Department of Medicine, Harvard Medical School, Boston, MA, USA; 3 Broad Institute of Harvard and Massachusetts Institute of Technology, Cambridge, MA, USA; 4 Division of Gastroenterology, Department of Medicine, University of Massachusetts Chan Medical School, Worcester, MA, USA; 5 James Tarpo Jr. and Margaret Tarpo Department of Chemistry, Purdue University, West Lafayette, IN, USA; 6 University of Technology Sydney, Sydney, New South Wales, Australia; 7 School of Materials Engineering, Purdue University, West Lafayette, IN, USA; 8 Borch Department of Medicinal Chemistry and Molecular Pharmacology, Purdue University, West Lafayette, IN, USA; 9 Purdue Institute for Cancer Research, Purdue University, West Lafayette, IN, USA

## Abstract

Mutations in isocitrate dehydrogenase 1 (IDH1) and 2 (IDH2) are common in multiple types of human cancer and cause accumulation of the oncometabolite D-2-hydroxyglutarate (D2HG) instead of α-ketoglutarate, driving cancers like gliomas and acute myeloid leukaemia by blocking cell differentiation and promoting tumour growth. Here we discovered protein *O*-2-hydroxyglutarylation by D2HG using chemical proteomics and further revealed distinct chiral preferences for D2HG and L-2-hydroxyglutarate (L2HG) modifications. D2HG modifications are upregulated in IDH-mutant cells or upon D2HG treatment, while L2HG modifications increase under hypoxic conditions or following L2HG treatment. Notably, two kinases MRCKA and SLK are modified by D2HG and L2HG, respectively, and confirmed by synthetic peptide standards. Phosphoproteomics revealed reduced phosphorylation of MRCKA and SLK substrates, suggesting crosstalk between D/L-2HG modification and kinase activity. These findings highlight distinctive roles of D/L-2HG modifications in cancer progression and suggest potential avenues for therapeutic targeting of oncometabolite-induced post-translational modifications.

Oncometabolites have come under the spotlight in recent years as pathognomonic hallmarks in human^1,2^. A prominent oncometabolite, D-2-hydroxyglutarate (D2HG) accumulates as a direct consequence of mutations in isocitrate dehydrogenase 1 or 2 (IDH1/2)^3–5^, which occur commonly in several cancer types like glioma and intrahepatic cholangiocarcinoma (ICC)^6–8^. These mutations confer a neomorphic activity that catalyses the reduction of α-ketoglutarate (α-KG) to D2HG in a NADPH-dependent manner, contributing to poor prognosis and reduced survival rates^9^. However, the precise molecular mechanisms remain incompletely understood.

The current view on the mechanism of IDH1/2 mutation holds that D2HG acts as an antagonist of α-KG to competitively inhibit the activity of α-KG-dependent dioxygenases, including those involved in histone and DNA demethylation^10,11^. Beyond these epigenetic alterations, recent studies have implicated D2HG in modulating key signalling pathways such as HIF-1, RTK and mTOR^12,13^. Moreover, previous studies also identified additional sources of D2HG from promiscuous enzyme reactions in cells lacking IDH mutations^10,11^. These studies have raised many questions regarding unknown function and activity of D2HG. Given the prevalence and importance of potential covalent modifications by cellular metabolites on proteins leading to distinct post-translational modifications (PTMs)^14,15^, investigating whether D2HG contributes to PTMs in IDH1/2-mutant cells is essential. This exploration opens up intriguing possibilities for developing therapeutic strategies targeting D2HG-related modifications.

Mass spectrometry (MS)-based proteomics has greatly advanced the study of PTMs, offering high sensitivity and throughput^15,16^. However, despite these technical strides, the identification of unique PTMs remains a formidable challenge. Traditional methods falter when faced with the inherent complexity of protein modifications, particularly when discovering previously unknown PTMs. The most direct approach for identifying PTMs involves analysing mass shifts of peptides in proteomic data through an open search^17–19^. However, this method is hindered by the inherently low sensitivity and abundance of modified peptides relative to their unmodified counterparts, making detection difficult and often elusive. While antibody-based enrichment can improve the detection of modified peptides, its effectiveness is highly dependent on the specificity and availability of suitable antibodies. Moreover, developing antibodies for previously uncharacterized PTMs is both challenging and time-consuming, limiting their utility for rapid discovery^15^. Given these obstacles, there is a pressing need for more efficient methods to discover PTMs, particularly in cancer research. Innovations in this area could provide deeper insights into the molecular mechanisms driving tumour progression and pave the way for therapeutic intervention discovery.

In this study, we report the discovery of covalent modifications by D2HG by chemical proteomics, employing a chemical affinity-based enrichment method and comparing human ICC cells with both wild-type and IDH1-mutant genotypes. Interestingly, we also discovered that its enantiomer L-2-hydroxyglutarate (L2HG), which accumulates under hypoxic conditions^11,20,21^, modifies a distinct set of proteins. By leveraging the unique properties of the enantiomers D2HG and L2HG, we revealed distinct protein groups modified by each metabolite, shedding light on molecular pathways that drive cancer progression. This study offers a powerful framework for investigating chirally dependent protein modifications (*O*-2-hydroxyglutarylation) in cancer, and lays the foundation for developing therapeutic strategies.

## Results

### Discovery of D2HG modifications by chemical proteomics

**Extended Data Fig. 1 |:**
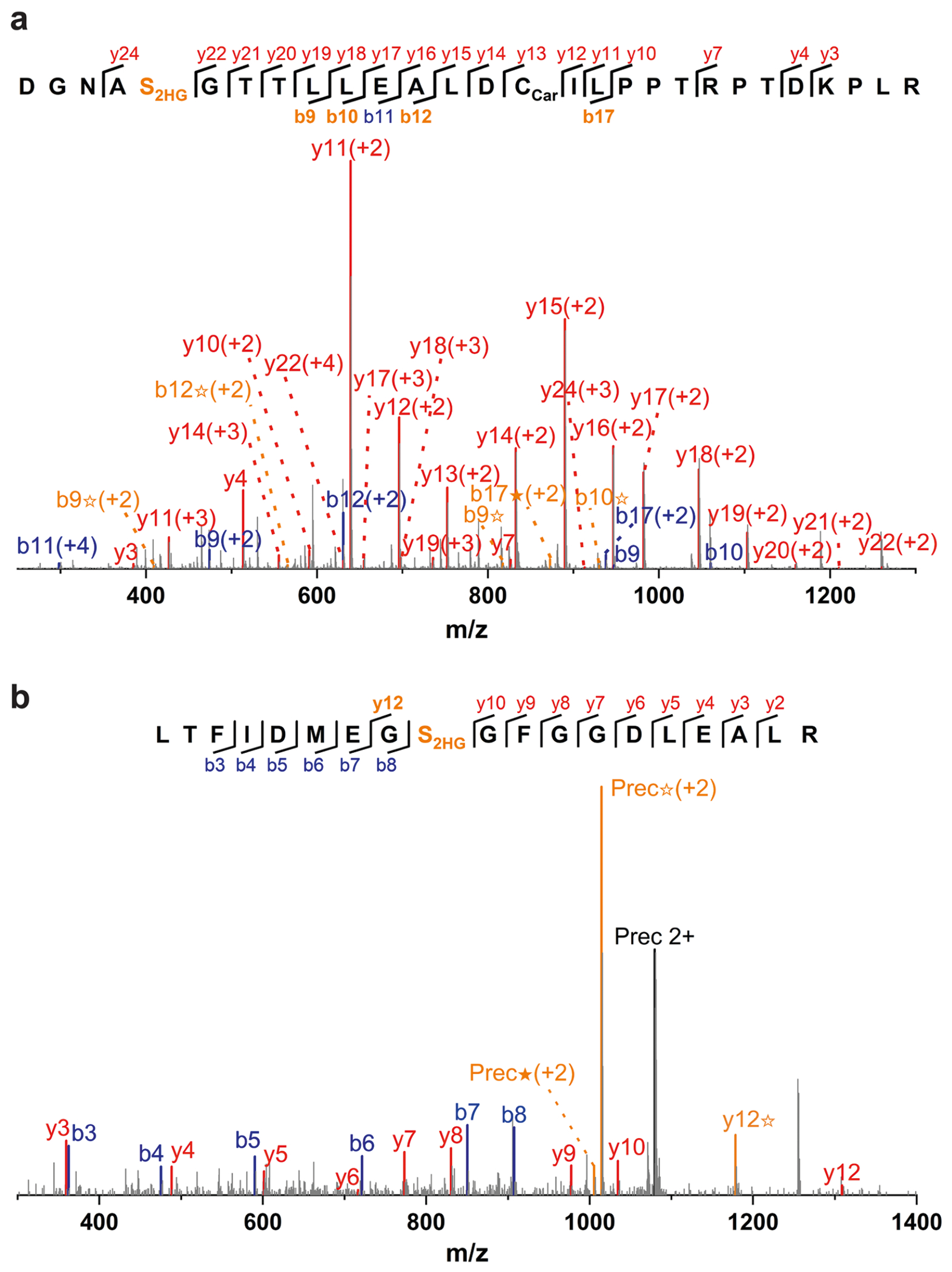
Representative MS/MS spectra of the 2HG modified peptides DGNAS_2HG_GTTLLEALDC_Car_ILPPTRPTDKPLR (a) and LTFIDMEGS_2HG_GFGGDLEALR (b). The b ion marked as blue refers to the N-terminal parts of the peptide, the y ion marked as red refers to the C-terminal parts of the peptide. Fragments showing neutral loss of 2HG are highlighted in bold orange, with ions from 2HG loss denoted by ★ and ions from (2HG–H_2_O) loss denoted by ✫.

To investigate potential covalent protein modifications by D2HG, we first conducted liquid chromatography (LC)–MS screening on mass shifts of tryptic peptides using an IDH1-mutant human ICC cell line, RBE cells, which produce elevated D2HG levels^22^. D2HG may covalently modify proteins by the formation of amide bonds on lysine, ester bonds on serine/threonine/tyrosine, thioester bonds through cysteine or ester bonds on aspartate/glutamate (Supplementary Fig. 1). Incorporating these possibilities as variable modifications in database searches and jointly analysing site localization across all peptides, we identified 308 modified sites from 138 peptides: 238 O-linked, 62 N-linked (Lys) and 8 S-linked (Cys) (Supplementary Table 1 and Extended Data Fig. 1a,b). These findings indicate that 2HG may modify a broad spectrum of residues, with *O*-linked modifications predominating. As including all seven residue types as variable modifications markedly expands the search space and increases false discovery—and given that PTMs on acidic residues are relatively uncommon—our focus was on *O*-2-hydroxyglutarylation of serine, threonine and tyrosine, as the *O*-modifications are dominant through the initial screening, but other linkage types remain to be further explored.

**Extended Data Fig. 2 |:**
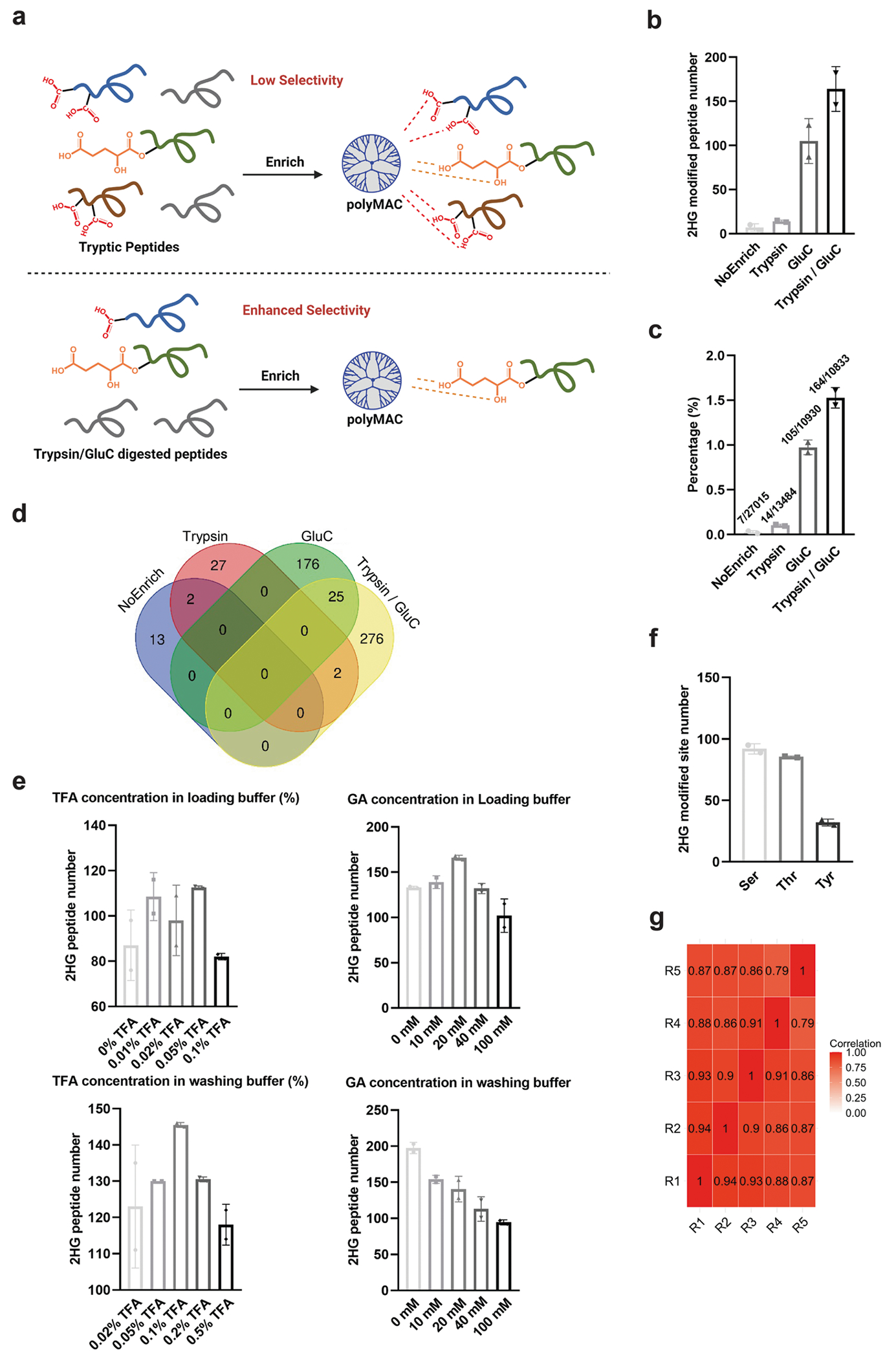
Investigation of polyMAC enrichment for 2HG modification. (**a**) Schematic representation of the polyMAC enrichment strategy for 2HG modifications. (**b**) Number of 2HG-modified peptides identified using different enrichment approaches (*n* = 2 technical replicates per group). (**c**) Proportion of 2HG-modified peptides among all peptides identified by different enrichment approaches (*n* = 2 technical replicates per group). (**d**) Venn diagram showing overlap and unique 2HG-modified sites across four digestion methods. (**e**) Method optimization for the polyMAC enrichment of 2HG-modified peptides digested by trypsin-GluC (*n* = 2 technical replicates per group). (**f**) Site distribution analysis for identified 2HG modified peptides (*n* = 2 technical replicates per group). (**g**) Reproducibility assessment of 2HG-modified peptide intensities across five technical replicates using Pearson correlation analysis. Pairwise Pearson correlation coefficients (r) were calculated between replicate intensities. Technical replicates were generated from the same cell lysate pool, which was divided into multiple aliquots and processed independently through the downstream workflow.

Our initial screening also revealed the low abundance and complexity of D2HG modifications and highlighted the need for enrichment of D2HG-modified proteins/peptides before LC–MS profiling. Antibody-based enrichment is effective but difficult to implement for low-abundance modifications owing to the need for prior knowledge on the sites and specificity. We therefore turned to polyMAC, which was originally developed for enriching phosphopeptides through Ti^4+^ ion–phosphate coordination^23^. Given that 2HG modifications introduce both hydroxyl and carboxyl groups, we reasoned they would display enhanced affinity for polyMAC (Extended Data Fig. 2a). We therefore tuned the enrichment protocol for D2HG-modified peptides and compared digestion strategies, including trypsin, GluC and sequential trypsin-GluC (optimization details in Supplementary Note 1). GluC digestion, which generates peptides with fewer acidic residues that can compete for polyMAC binding, improved detection relative to trypsin alone, while sequential trypsin-GluC digestion gave the highest yield and selectivity for 2HG-modified peptides (Extended Data Fig. 2b,c and Supplementary Table 2). A Venn diagram illustrates the overlap and unique sites across the four digestion strategies (Extended Data Fig. 2d). The minimal overlap between the trypsin-only and trypsin-GluC datasets probably arises from the distinct peptide pools generated by different cleavage specificities, which in turn affect enrichment and LC–tandem MS detectability. In silico GluC cleavage of the tryptic 2HG-modified peptides indicated that several would become too short (<7 amino acids) and many would lose basic residues, markedly reducing positive charge, which explains their absence after the sequential digest. Also, the relatively low specificity for 2HG-modified peptides makes the identification more susceptible to competition from unmodified peptides. Although trypsin alone yielded some unique sites, we did not include the trypsin-only approach to expand coverage because its low specificity may increase the risk of false positives. After reoptimizing conditions for the sequential workflow, we identified approximately 200 2HG-modified peptides in a single LC–MS run (for details see Supplementary Note 1 and Extended Data Fig. 2e). Analysis of 2HG modification sites revealed a serine>threonine>tyrosine distribution, similar to phosphorylation patterns^24^, suggesting potential crosstalk between these PTMs (Extended Data Fig. 2f and Supplementary Table 3).

**Fig. 1 |:**
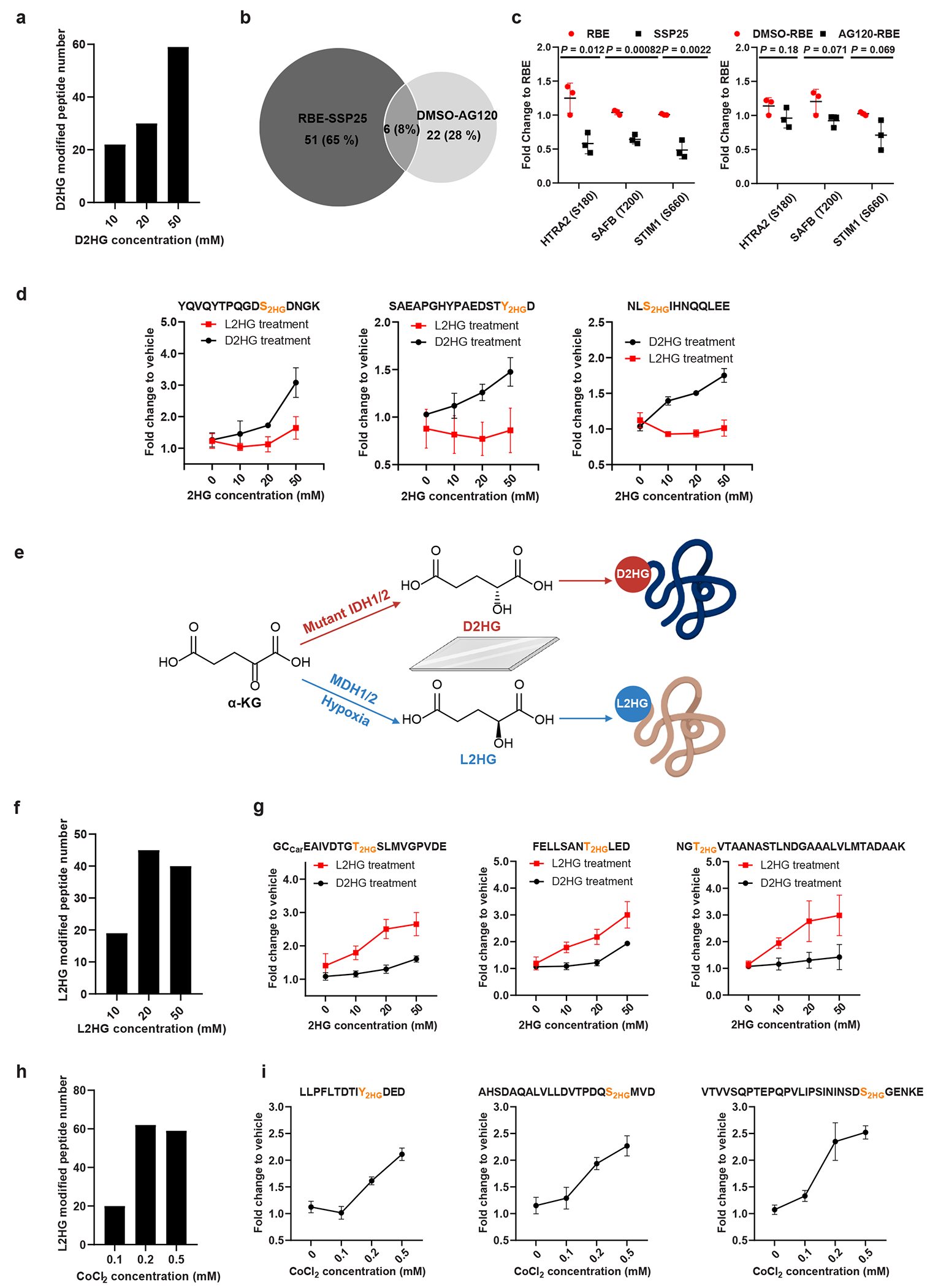
Chirally dependent D2HG and L2HG modifications on proteins. **a**, Identification of D2HG-modified peptides through quantitative proteomics. H293T cells were treated with different D2HG concentrations for 24 hours. **b**, Overlap analysis of the D2HG-modified peptides identified by quantitative proteomics. The differential analysis was performed between SSP25 and RBE cells, as well as AG120-treated and DMSO-treated RBE cells. **c**, Peak area fold changes of representative D2HG-modified peptides in SSP25 versus RBE cells (relative to RBE cells), and in AG120-treated versus DMSO-treated RBE cells (relative to DMSO-treated RBE cells). **d**, Peak area fold changes of representative D2HG-modified peptides in H293T cells treated with a gradient concentration of D2HG and L2HG, relative to vehicle control. **e**, Scheme for D2HG and L2HG modification on specific proteins in a chirality behaviour. **f**, Identification of L2HG-modified peptides via quantitative proteomics. H293T cells were treated with different L2HG concentrations for 24 hours. **g**, Peak area fold changes of representative L2HG-modified peptides in H293T cells treated with a gradient concentration of D2HG and L2HG, relative to vehicle control. **h**, Identification of L2HG-modified peptides via quantitative proteomics. H293T cells were treated with different CoCl_2_ concentrations for 24 hours. **i**, Peak area fold change of representative L2HG-modified peptides in H293T cells treated with a gradient concentration of CoCl_2_, relative to vehicle control. For quantitative proteomics (**a–c,f,h**), the peptides with a fold change >1.2 and *P* value <0.05 in the D2HG (or L2HG) accumulated groups compared with the non-accumulated group were considered as D2HG (or L2HG)-modified peptides. Peptide abundance was calculated from peak areas normalized to the total ion current (TIC). Data represent mean ± s.d. (*n* = 3 biological replicates per group). Two-tailed *t*-tests were used to calculate *P* values without adjustment. Schematics in **e** created in BioRender; Qu, Z. https://biorender.com/jk21a5m (2026).

MS-based PTM identification relies on detecting fixed mass shifts in peptide precursor ions and tandem MS (MS/MS) fragment ions. However, metabolites with the same chemical formula as D2HG can theoretically generate this same 130.0266-Da shift in LC–MS. Supplementing cells with exogenous metabolites is known to elevate intracellular concentrations, potentially leading to a corresponding increase in metabolite-mediated modifications. To explore this, we treated H293T cells with a gradient concentration of D2HG, followed by cell lysis, protein extraction and digestion. Peptides with D2HG modification were enriched using polyMAC and analysed by LC–MS with label-free quantitation. Peptides exhibiting a fold change >1.2 and a *P* value <0.05 in D2HG-treated groups compared to control groups were classified as D2HG-modified. Compared with PBS-only controls, D2HG-treated cells showed a dose-dependent increase in cellular D2HG concentration (Supplementary Fig. 2a) and D2HG-modified peptides (Fig. 1b and Supplementary Table 4). Time-dependent treatment also increased identification of D2HG-modified peptides (Supplementary Fig. 2b and Supplementary Table 5).

**Extended Data Fig. 3 |:**
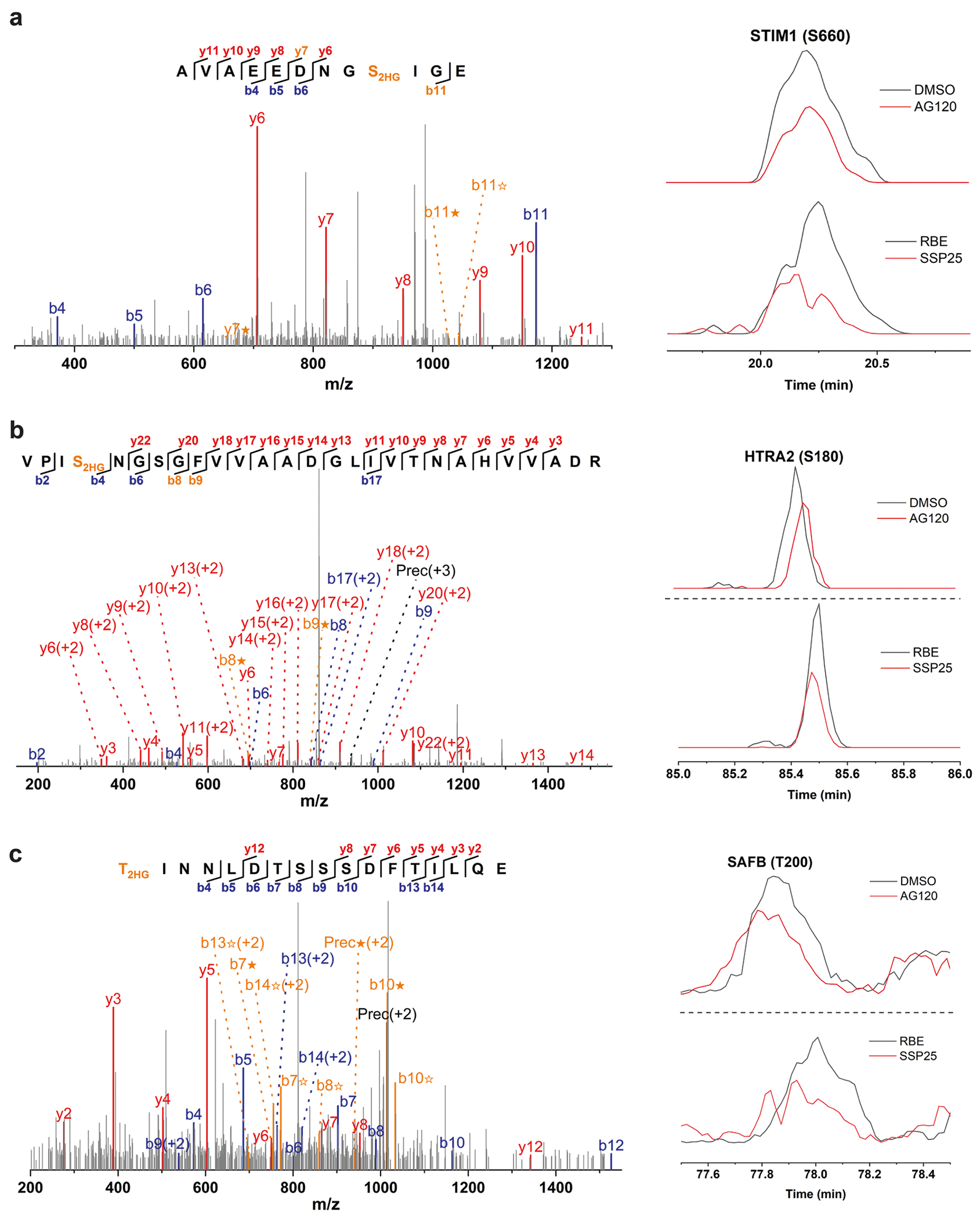
MS/MS spectra and EICs of representative D2HG modified peptides derived from RBE cells, SSP25 cells and RBE cells treated with or without AG120. The peptide sequences are as follows: (**a**) AVAEEDNGS_2HG_IGE; (**b**) VPIS_2HG_NGSGFVVAADGLIVTNAHVVADR; (**c**) T_2HG_INNLDTSSSDFTILQE. The b ion marked as blue refers to the N-terminal parts of the peptide, the y ion marked as red refers to the C-terminal parts of the peptide. Fragments showing neutral loss of 2HG are highlighted in bold orange, with ions from 2HG loss denoted by ★ and ions from (2HG–H_2_O) loss denoted by ✫. Data represent three biologically independent repeats.

IDH1-mutant cells can produce D2HG at markedly higher levels compared to wild-type cells^7,25^, and AG120 is able to inhibit D2HG synthesis in IDH1-mutant cells^26,27^ (Supplementary Fig. 2c). We reason that the abundance of D2HG modifications should theoretically correlate with fluctuations in endogenous D2HG levels. Through quantitative proteomics, we examined D2HG modifications in wild-type SSP25 cells, IDH1-mutant RBE cells and AG120-treated IDH1-mutant cells. In total, we identified 79 D2HG-modified peptides (Fig. 1b and Supplementary Table 6). Many of these peptides showed reduced abundance following AG120 treatment (Fig. 1c and Extended Data Fig. 3). Notably, these D2HG-modified proteins include HTRA2, SAFB and STIM1. HTRA2, a serine protease involved in apoptosis^28^, was modified at S180 within its catalytic domain, suggesting potential impacts on activity. STIM1, a Ca^2+^ sensor linked to tumour metastasis and poor prognosis^29^, and SAFB a negative regulator of proliferation^30^, were also modified. All these proteins are involved in tumour progression, suggesting that D2HG modifications may play a regulatory role in cancer development.

### Chiral preference of D2HG and L2HG in modifying proteins

**Extended Data Fig. 4 |:**
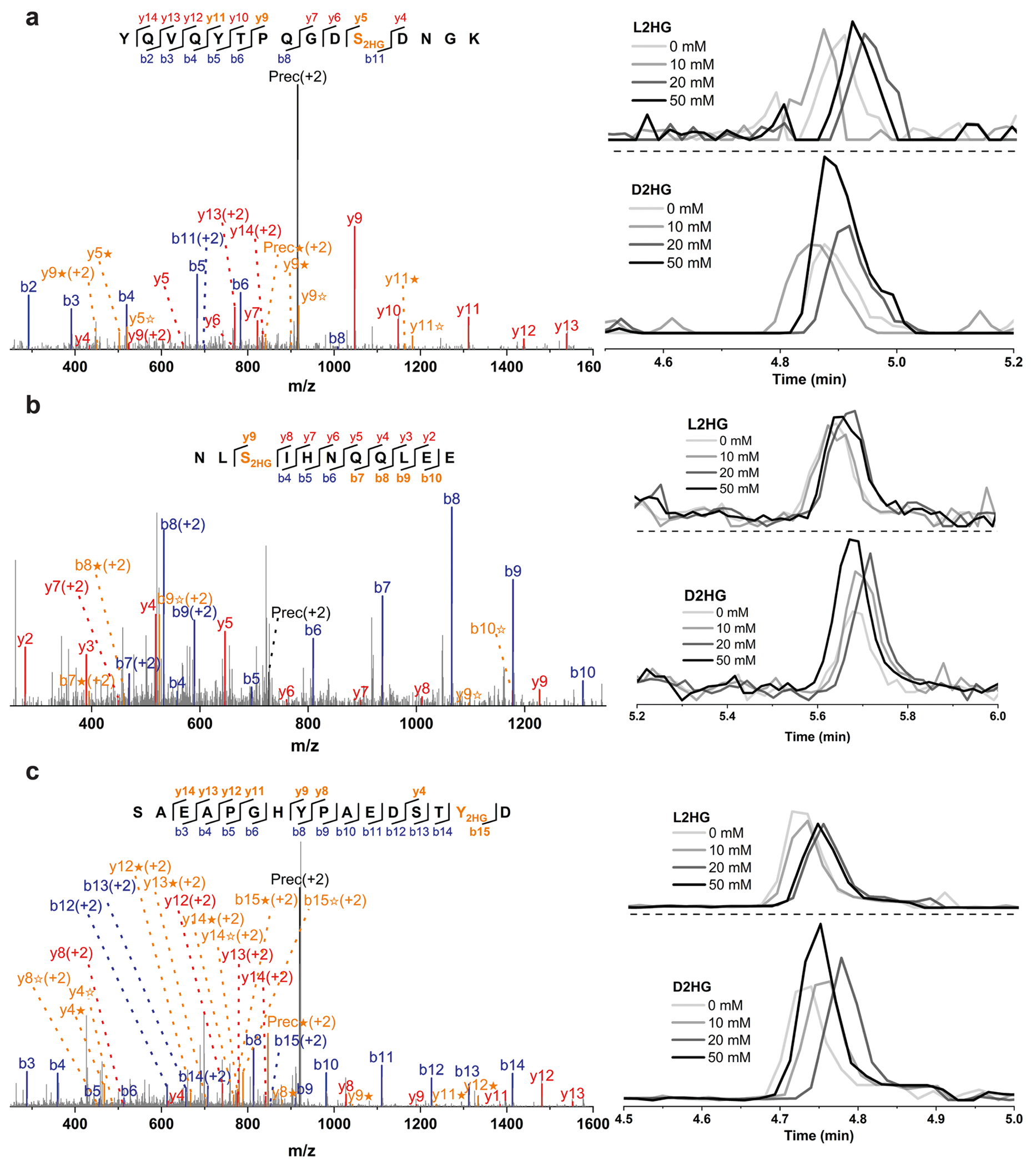
MS/MS spectra and EICs of representative D2HG modified peptides detected in H293T cells treated with exogenous D2HG and L2HG. The peptide sequences are as follows: (**a**) YQVQYTPQGDS_2HG_DNGK; (**b**) NLS_2HG_IHNQQLEE; (**c**) SAEAPGHYPAEDSTY_2HG_D. The b ion marked as blue refers to the N-terminal parts of the peptide, the y ion marked as red refers to the C-terminal parts of the peptide. Fragments showing neutral loss of 2HG are highlighted in bold orange, with ions from 2HG loss denoted by ★ and ions from (2HG–H_2_O) loss denoted by ✫. Data represent three biologically independent repeats.

Inspired by recent research on mirror-image biology^31^ and enantiomeric preferences of chemical probes^32^, we tested whether D2HG modifications are chiral dependent by treating cells with D2HG and L2HG in parallel. For proteins showing dose-dependent 2HG modifications with D2HG treatment, L2HG treatment did not markedly increase modifications, indicating that the effect is primarily due to D2HG and is indeed chirality dependent (Fig. 1d and Supplementary Tables 4 and 7). Corresponding chromatograms and MS/MS spectra of representative peptides are shown in Extended Data Fig. 4.

**Extended Data Fig. 5 |:**
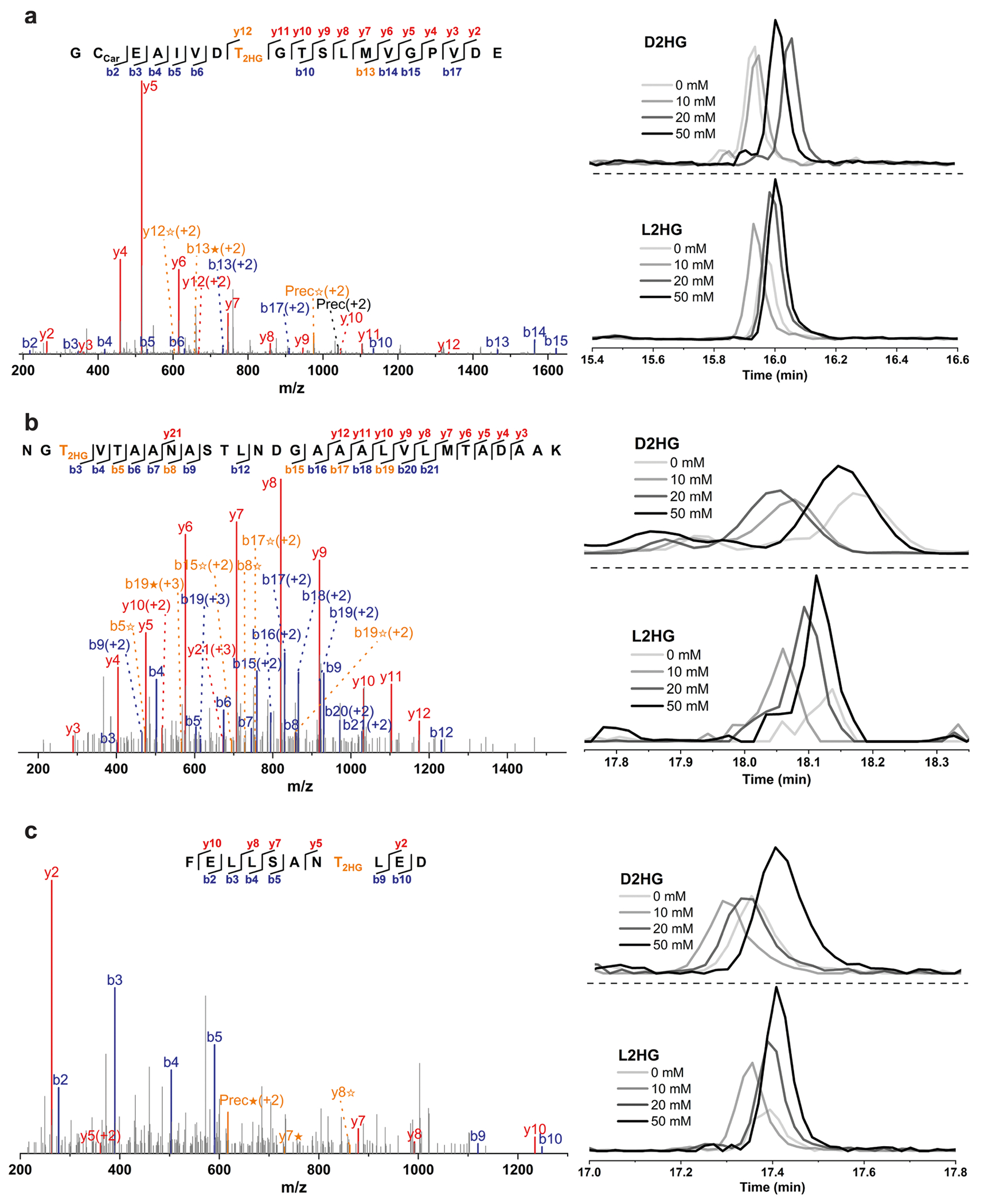
MS/MS spectra and EICs of representative L2HG modified peptides detected in H293T cells treated with exogenous D2HG and L2HG. The peptide sequences are as follows: (**a**) GC_Car_EAIVDT_2HG_GTSLMVGPVDE; (**b**) NGT_2HG_VTAANASTLNDGAAALVLMTADAAK; (**c**) FELLSANT_2HG_LED. The b ion marked as blue refers to the N-terminal parts of the peptide, the y ion marked as red refers to the C-terminal parts of the peptide. Fragments showing neutral loss of 2HG are highlighted in bold orange, with ions from 2HG loss denoted by ★ and ions from (2HG–H_2_O) loss denoted by ✫. Data represent three biologically independent repeats.

We observed some peptides with mass shifts similar to D2HG, but their levels did not correlate with exogenous D2HG treatment concentrations. We hypothesize that some of these peptides may originate from L2HG (Fig. 1e). To validate this, we treated cells with different concentrations of L2HG. L2HG treatment led to increases in cellular L2HG levels (Supplementary Fig. 3a) and, interestingly, parallel increase in the identification of L2HG-modified peptides by quantitative proteomics analysis (Fig. 1f and Supplementary Table 7). Prolonged L2HG treatment produced similar effects (Supplementary Fig. 3b and Supplementary Table 8). Three representative L2HG-modified peptides, which increased noticeably with L2HG compared to D2HG treatment, suggest these modifications are primarily L2HG-induced (Fig. 1g and Extended Data Fig. 5).

**Extended Data Fig. 6 |:**
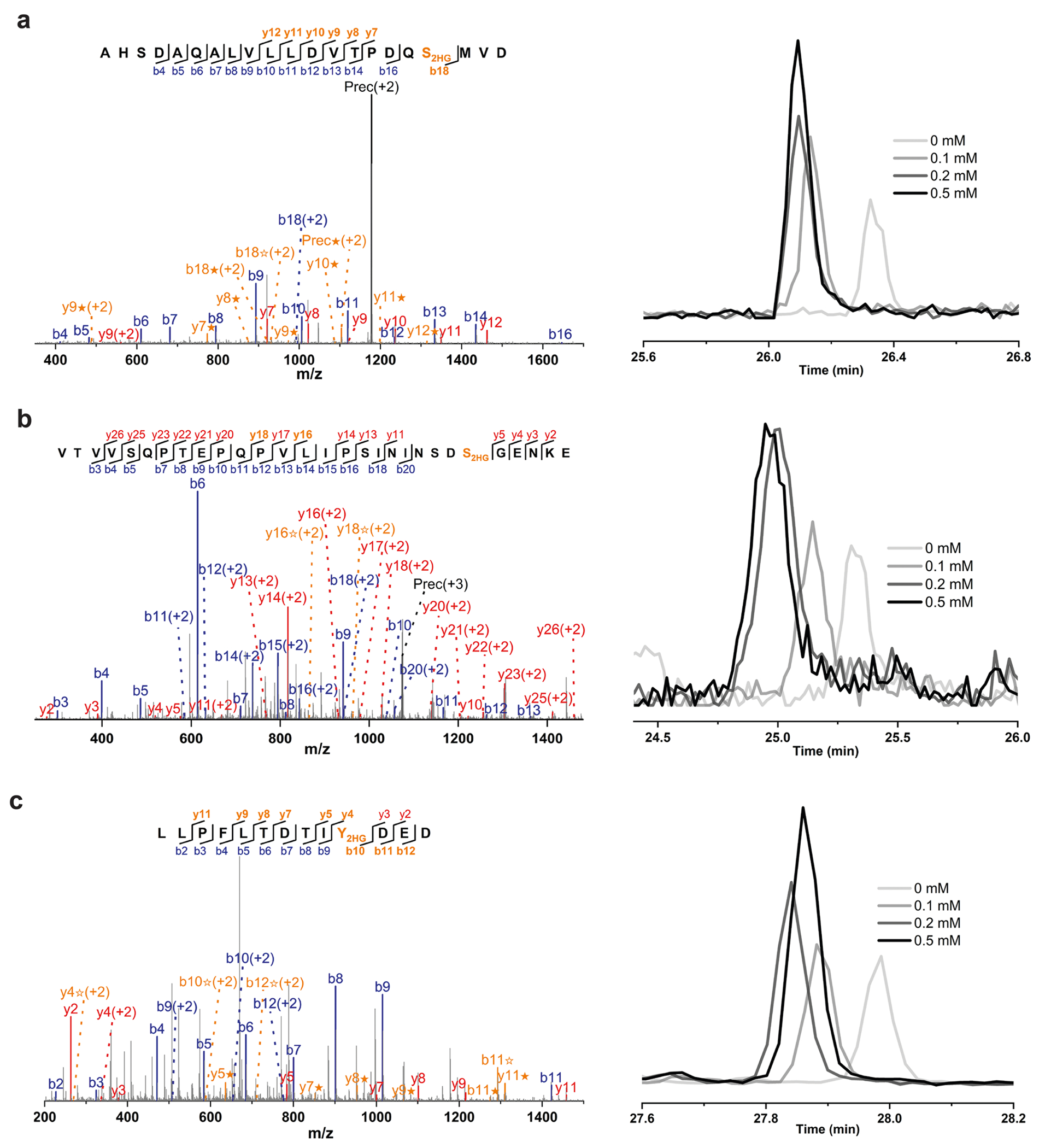
MS/MS spectra and EICs of representative L2HG modified peptides detected in H293T cells treated with CoCl2. The peptide sequences are as follows: (**a**) AHSDAQALVLLDVTPDQS_2HG_MVD; (**b**) VTVVSQPTEPQPVLIPSININSDS_2HG_GENKE; (**c**) LLPFLTDTIY_2HG_DED. The b ion marked as blue refers to the N-terminal parts of the peptide, the y ion marked as red refers to the C-terminal parts of the peptide. Fragments showing neutral loss of 2HG are highlighted in bold orange, with ions from 2HG loss denoted by ★ and ions from (2HG–H_2_O) loss denoted by ✫.

**Extended Data Fig. 7 |:**
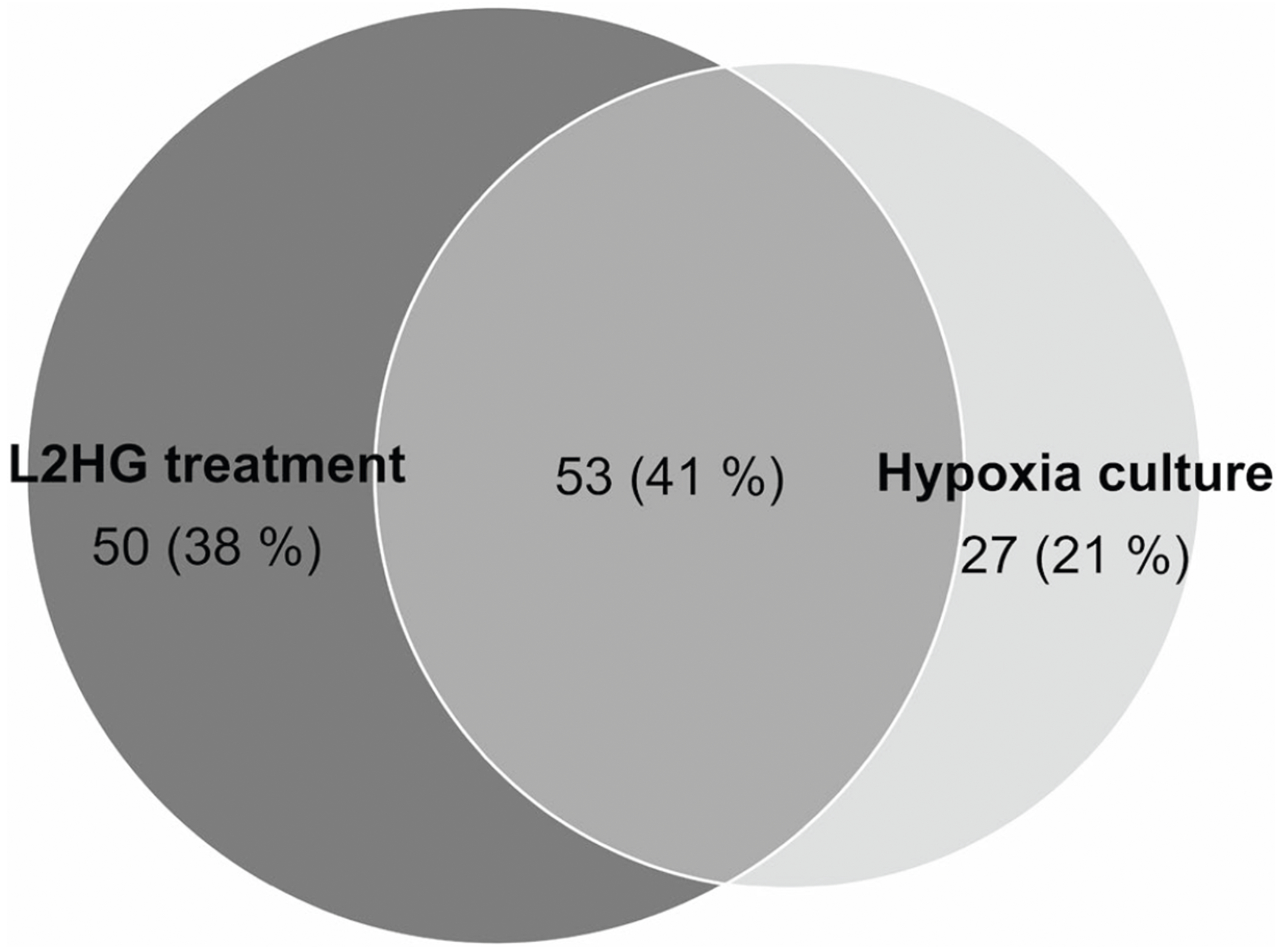
Comparison of L2HG-modified peptides identified from exogenous L2HG-treated and hypoxia-cultured H293 cells. The peptides with a fold change > 1.2 and *P* < 0.05 in the L2HG accumulated groups compared with the non-accumulated group were considered as L2HG modified peptides, while the peptides with fold change < 0.833 and *P* < 0.05 were placed on a blacklist and excluded (*n* = 3 biological replicates per group). Two-tailed t-tests were used to calculate *P* values without adjustment. The detailed screening and filtering workflow is described in Supplementary Note 2.

It has been reported that the levels of the L2HG are increased by changes in the tumour microenvironment, including hypoxia and a low pH^21^. By exposing H293T cells to varying concentrations of the hypoxia inducer CoCl_2_ (0.1–0.5 mM), we observed a parallel increase in the abundance of endogenous L2HG (Supplementary Fig. 3c) and L2HG-modified peptides by quantitative proteomics (Fig. 1h and Supplementary Table 9), with three representative peptides correlating with CoCl_2_ levels (Fig. 1i and Extended Data Fig. 6). Similar results were observed when cells were cultured in a hypoxia chamber with 2% oxygen (Supplementary Fig. 3d and Supplementary Table 10). An evident overlap between L2HG-modified peptides identified in exogenous L2HG-treated cells and hypoxia-cultured cells supports a close correspondence between exogenous and endogenous L2HG accumulation (Extended Data Fig. 7). These findings underscore hypoxia-induced upregulation of L2HG modifications, suggesting their potential role in critical regulatory mechanisms within the hypoxic tumour microenvironment.

### Validation of D/L-2HG modifications

**Fig. 2 |:**
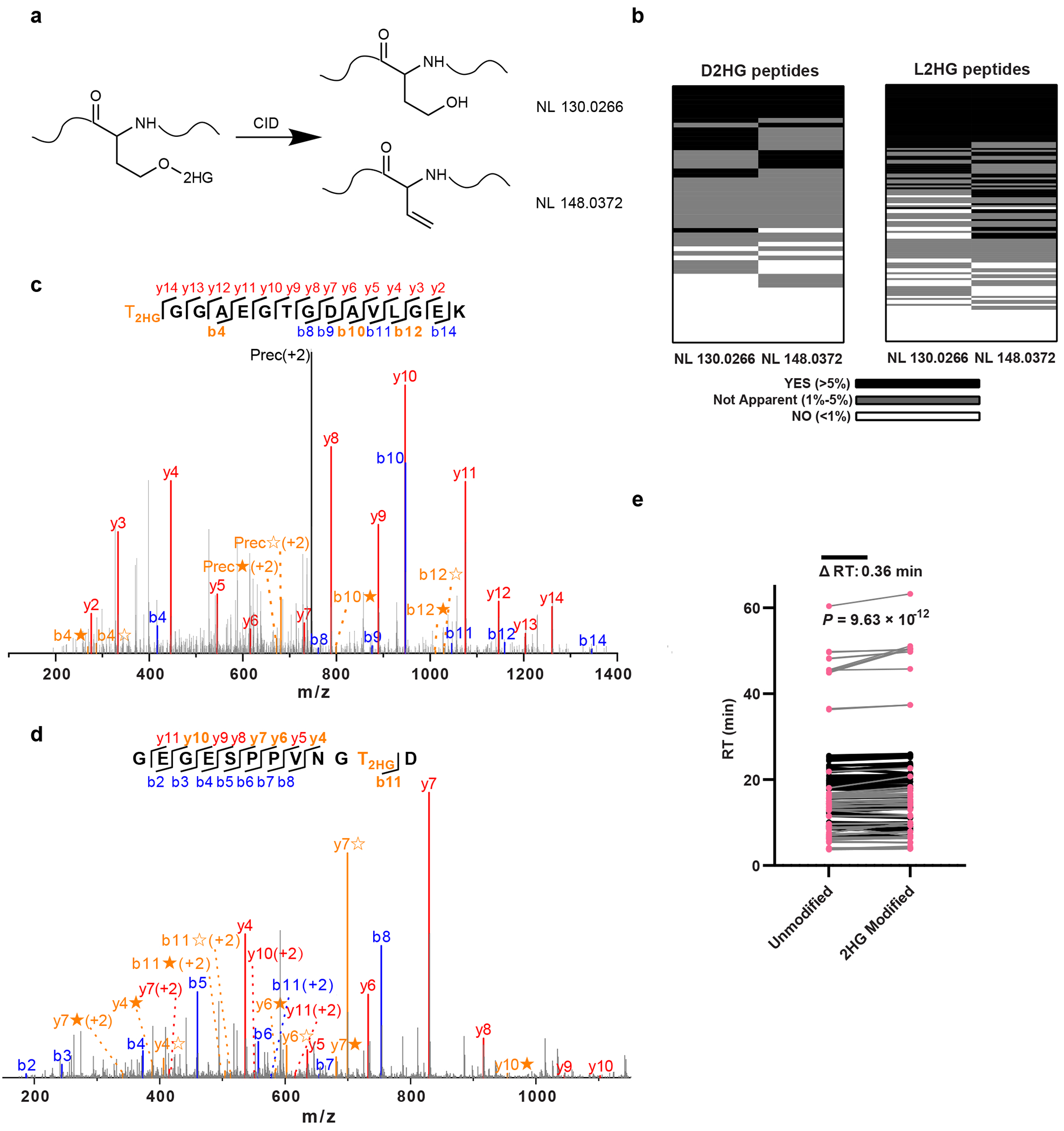
Chromatographic and MS/MS fragmentation analysis of 2HG-modified peptides. **a**, Schematic representation of diagnostic neutral loss ions generated from 2HG-modified peptides during MS/MS fragmentation. **b**, Frequency of neutral loss ions observed among assigned 2HG-modified peptides. MS/MS spectra from 56 high-confidence D2HG-modified peptides and 130 high-confidence L2HG-modified peptides were analysed. High-confidence peptides were defined based on cross-validation across the 20 dataset groups (three biological replicates per group) using the filtering workflow described in Supplementary Note 2. Neutral loss ion abundances >5% are shown in black, 1–5% in grey and <1% in white. **c**,**d**, Representative MS/MS spectra of the 2HG-modified peptides T_2HG_GGAEGTGDAVLGEK (**c**) and GEGESPPVNGT_2HG_D (**d**). The b ions are shown in blue and the y ions are shown in red. The neutral loss ions are highlighted in orange, with ions from 2HG loss denoted by ★ and ions from (2HG–H_2_O) loss denoted by ✫. **e**, Pairwise comparison of retention times between 2HG-modified peptides and their corresponding unmodified counterparts (*n* = 235 peptide pairs from 20 dataset groups, three biological replicates per group). The *P* value was calculated by a paired two-tailed Student’s *t*-test without adjustment.

Molecular characterization, such as MS/MS fragmentation patterns, provide critical evidence for the existence of PTMs. To minimize false positives and confirm the presence of D2HG-modified or L2HG-modified peptides, we focused on the neutral loss ions typically associated with ester modifications, such as phosphorylation, ADP-ribosylation and sulfation^33,34^. For D2HG and L2HG, 130.0266 Da and 148.0372 Da neutral loss ions from ester bond cleavage were observed (Fig. 2a). Manual inspection for the detected D2HG-modified or L2HG-modified peptides confirmed that most modified peptides exhibited neutral loss ions, although some at low abundance (Fig. 2b). Two representative MS/MS spectra display clear b/y ions and neutral loss ions (Fig. 2c,d), collectively validating D2HG and L2HG modifications. These neutral losses thus serve as useful validation markers, although they are not definitive proof of *O*-2-hydroxyglutarylation, as isomeric modifications could produce similar patterns. Comparison of 2HG-modified and unmodified peptides revealed increased retention times for modified peptides on reversed phase columns (Fig. 2e). Calculated log*P* values by ACD/Labs showed higher hydrophobicity in 2HG-modified serine (−0.97 ± 0.45) than in unmodified serine (−1.58 ± 0.33), which aligns with observed retention shifts and could serve as an additional indicator of 2HG modifications.

**Extended Data Fig. 8 |:**
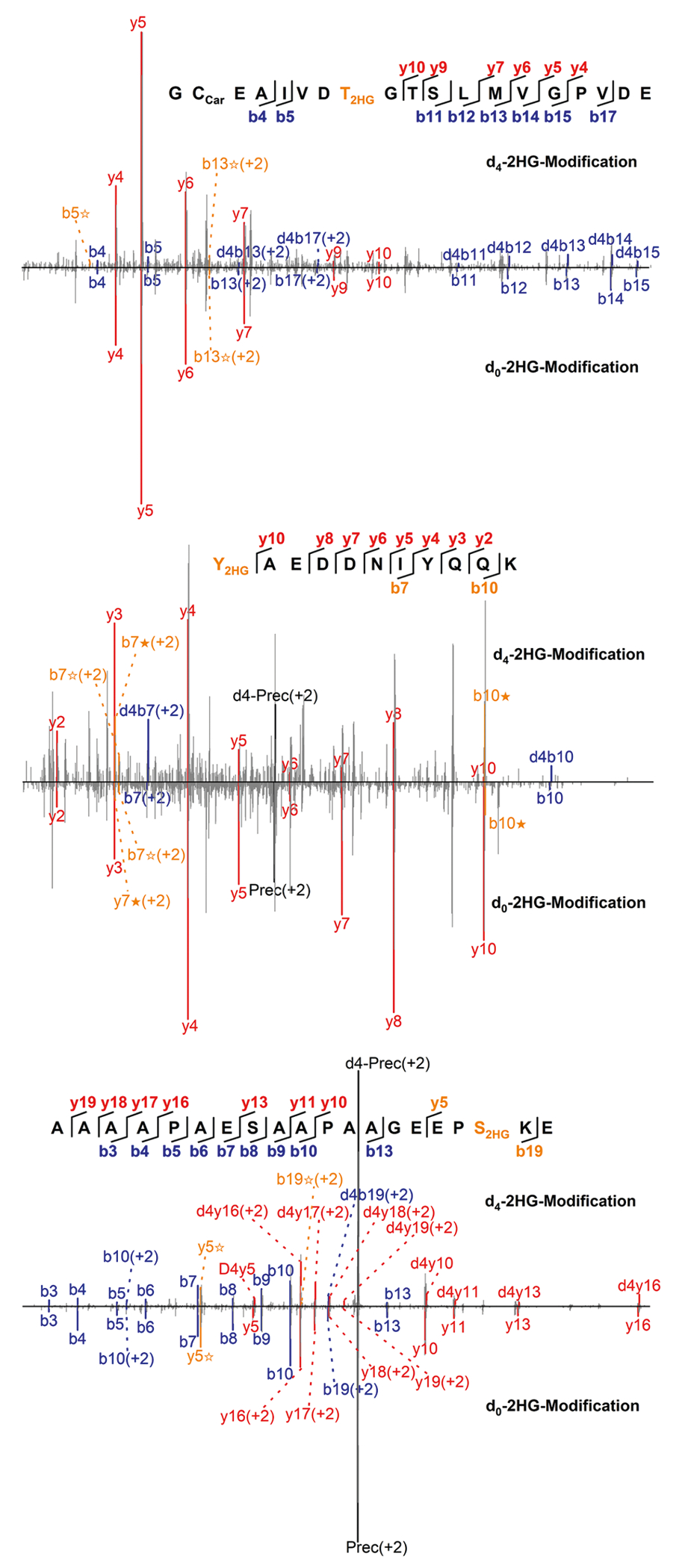
Representative MS/MS spectra of 2HG modified peptides and d4-2HG isotopically labelled versions. The b ion marked as blue refers to the N-terminal parts of the peptide, the y ion marked as red refers to the C-terminal parts of the peptide. Fragments showing neutral loss of 2HG are highlighted in bold orange, with ions from 2HG loss denoted by ★ and ions from (2HG–H_2_O) loss denoted by ✫.

To further enhance the specificity and validate our findings on D2HG-modified and L2HG-modified peptides, we used isotopic metabolic flux analysis by incubating cells with isotopically labelled d_4_-D/L-2HG to directly track potential incorporation of isotopic 2HG tag. The isotopic metabolic flux experiment allowed us to identify 124 d_4_-2HG-modified peptides (Supplementary Table 11). MS/MS spectra of heavy-labelled and unlabelled 2HG-modified peptides showed identical fragmentation with a +4-Da shift, confirming endogenous 2HG modification structure (Extended Data Fig. 8).

**Fig. 3 |:**
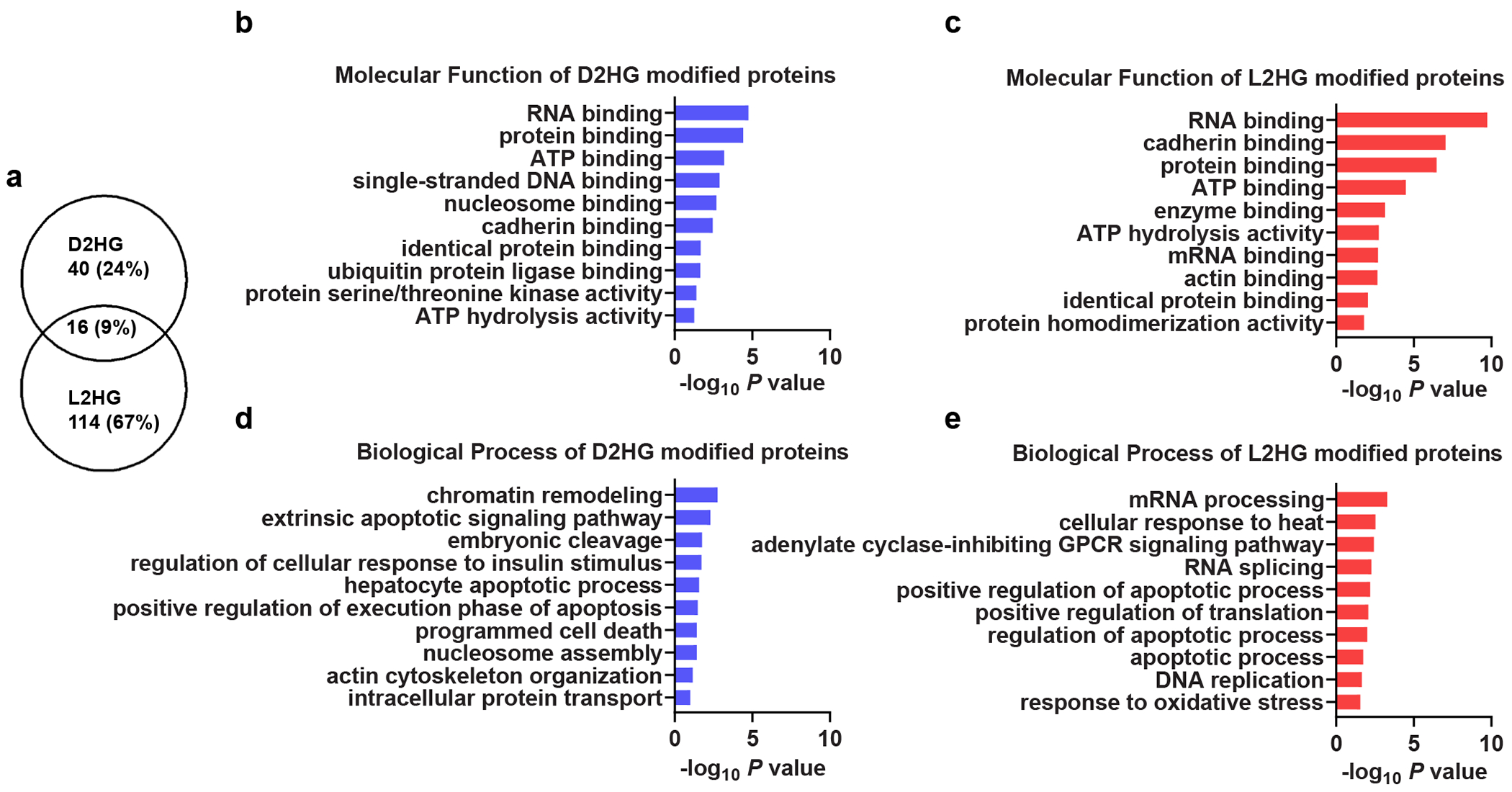
Analysis of D2HG and L2HG modifications. **a**, Overlap analysis of high-confidence D2HG-modified (*n* = 56) and L2HG-modified (*n* = 130) peptides. **b**–**e**, GO enrichment analysis of proteins specifically modified by D2HG (**b**,**d**) or L2HG (**c**,**e**). GO analysis was performed using DAVID, and the top 10 enriched terms are shown for biological process (**d**,**e**) and molecular function (**b**,**c**) categories. The input protein lists consisted of 40 high-confidence D2HG-specific and 114 high-confidence L2HG-specific modified proteins. High-confidence peptides were defined based on cross-validation across the 20 dataset groups (three biological replicates per group) using the filtering workflow described in Supplementary Note 2. *P* values were calculated in DAVID using a modified Fisher’s exact test (EASE score) without adjustment.

Overall, quantitative PTM analysis yielded 56 high-confidence D2HG-modified peptides in D2HG-accumulated cells (the criteria for high-confidence identification are detailed in Supplementary Note 2), showing strong overlap between IDH1-mutant RBE and H293T cells, and 130 high-confidence L2HG-modified peptides in L2HG-accumulated cells (Supplementary Tables 12 and 13). Distinct modification patterns were observed, with 40 D2HG-specific and 114 L2HG-specific peptides (Fig. 3a). These differences probably reflect stereospecific binding preferences and may underlie divergent biological functions. Gene ontology (GO) analysis supported this view: D2HG-specific proteins were enriched in chromatin remodelling and nucleosome binding consistent with D2HG’s role in histone and genome regulation^9^, whereas L2HG-specific proteins were linked to oxidative stress responses, which are known to be related to hypoxia-induced L2HG accumulation^11^ (Fig. 3b–e). These findings suggest that, in addition to the direct roles of D/L-2HG metabolites, protein modifications by D2HG and L2HG may contribute to the regulation of these biological processes.

We detected 18 shared sites across 16 peptides modified by both D2HG and L2HG, including MCM4/5 (DNA-replication licensing^35^) and XRCC1 (a base-excision repair scaffold^36^), reinforcing links between 2HG and impaired DNA repair/genome instability. RSBN1 (KDM9)—previously a non-covalent D2HG interactor^37^—was covalently modified by both enantiomers, suggesting effects on demethylase-mediated chromatin control. In addition, D/L-2HG mimics α-KG and can bind α-KG-dependent enzymes. We also identified three α-KG-dependent enzymes modified by L2HG, including AASS (inhibited by L2HG^38^), PHGDH (capable of promiscuous 2HG generation^11^) and GFPT1. These results suggest that 2HG modification adds a covalent regulatory layer to metabolic rewiring in cancer.

### D2HG and L2HG modification inhibit activity of select kinases

**Fig. 4 |:**
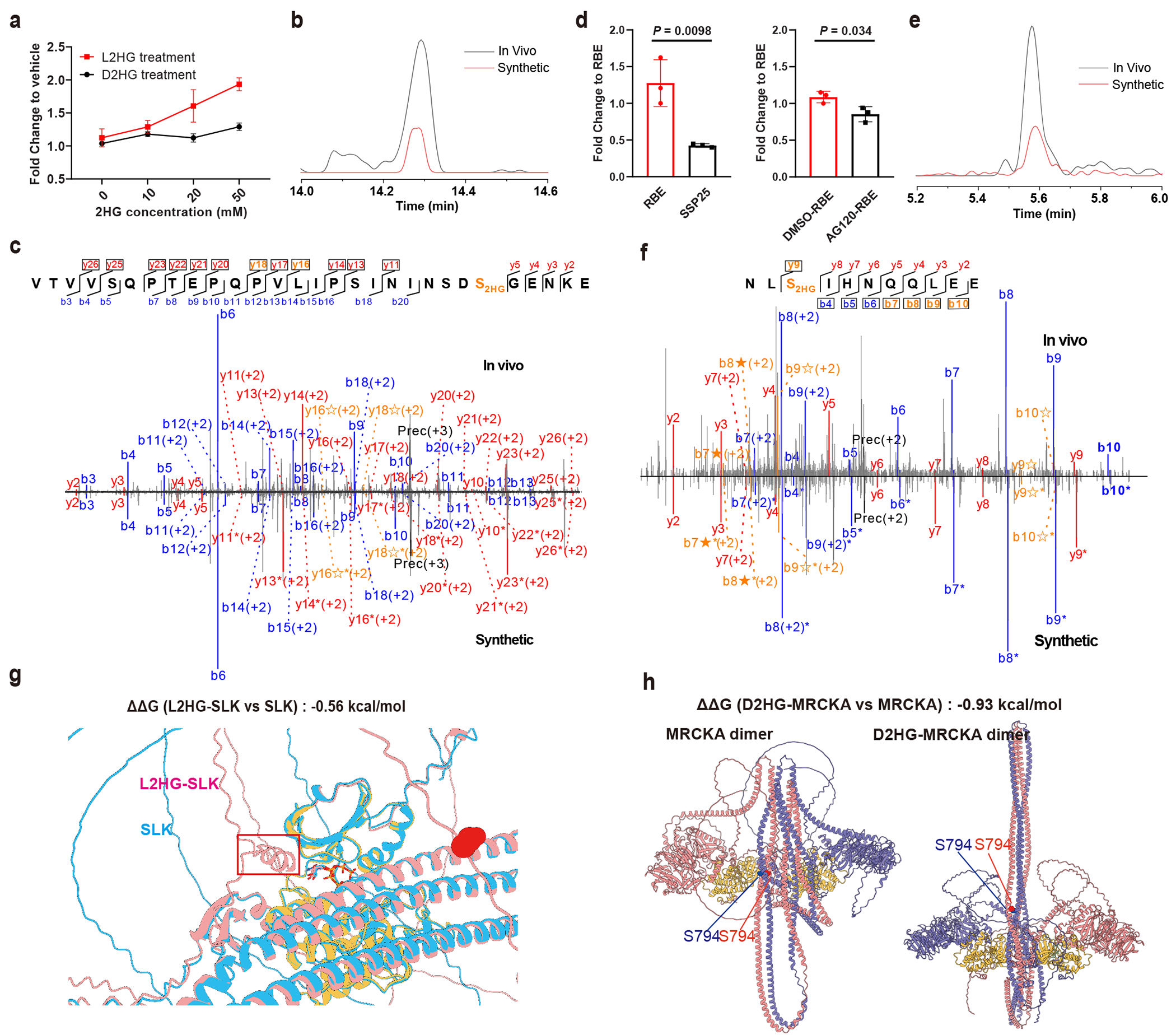
Kinase SLK modified with L2HG and MRCKA modified with D2HG possess decreased activity. **a**, Peak area fold changes of L2HG-modified SLK (S719) in H293T cells treated with a gradient concentration of D2HG and L2HG, relative to vehicle control. **b**,**c**, Comparison of extracted ion chromatograms (EICs) (**b**) and MS/MS spectra (**c**) between in vivo-detected peptide and synthetic isotopic standard for SLK (S719). The isotopic standard was generated with DL-serine (^13^C_3_,^15^N) coupled to 2HG. **d**, Peak area fold changes of D2HG-modified MRCKA (S794) in SSP25 versus RBE cells (relative to RBE cells), and in AG120-treated versus DMSO-treated RBE cells (relative to DMSO-treated RBE cells). **e**,**f**, Comparison of EICs (**e**) and MS/MS (**f**) spectra between in vivo-detected peptide and synthetic isotopic standard for MRCKA (S794), generated with DL-serine (^13^C_3_,^15^N) coupled to 2HG. **g**, Structures of the SLK with or without L2HG modification. Light blue represents SLK, pink represents L2HG-SLK and yellow represents active pocket. **h**, Structures of the MRCKA dimer with or without D2HG modification. Yellow represents active pocket. For MS/MS spectra (**c**,**f**): b ions (blue) correspond to N-terminal fragments, y ions (red) to C-terminal fragments. Fragments showing neutral loss of 2HG are highlighted in bold orange, with ions from 2HG loss denoted by ★ and from (2HG–H_2_O) loss denoted by ✫. Fragments containing the heavy serine are denoted by *. Peptide abundance was calculated from peak areas normalized to the TIC. Data represent mean ± s.d. (*n* = 3 biological replicates per group). Two-tailed *t*-tests were used to calculate *P* values without adjustment.

GO analysis (Fig. 3b) revealed 2HG-modified proteins associated with kinase signalling. To investigate the effect of 2HG modifications on kinases, we manually examined all 2HG-modified kinases in our data. As representative, 2HG modification was observed at Ste20-like serine/threonine kinase (SLK) S719, with levels increasing following exogenous L2HG treatment, while D2HG had little effect (Fig. 4a). CoCl_2_ treatment in H293T cells also elevated L2HG-modified SLK, correlating with cellular L2HG levels (Fig. 1i, right and Supplementary Fig. 3c). To confirm this site, we synthesized isotopically labelled peptides containing heavy serine at S719. Comparison of diagnostic isotopic b/y ions, retention times, and fragmentation patterns between synthetic standards and in vivo-detected peptides verified the SLK S719 modification (Fig. 4b,c).

**Extended Data Fig. 9 |:**
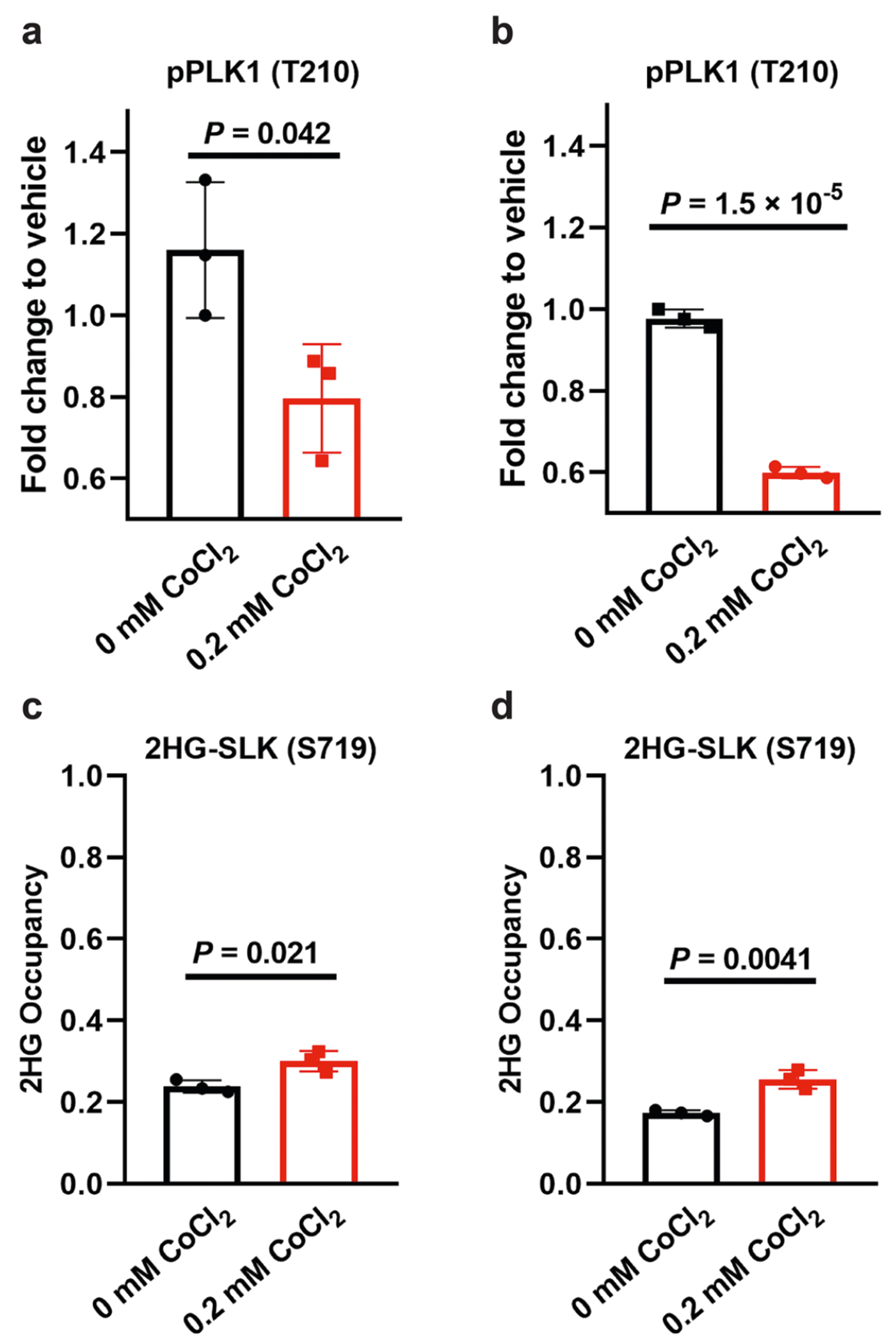
Quantitative analysis of L2HG modification and SLK activity. (**a,b**) MS^1^ TIC-normalized peak area fold changes of phosphorylated PLK1 (T210) in H293 cells (**a**) and in the in-vitro kinase assay (**b**), relative to vehicle control. (**c,d**) 2HG modification occupancy of SLK in H293 cells (**c**) and in purified His-SLK (**d**). Modification occupancy was calculated by comparing the peak areas of modified and unmodified SLK (S719) detected in the same LC–MS run, using the formula: *f*_mod_ = *A*_mod_/(*A*_mod_ + *A*_unmod_). Data represent mean ± s.d. (*n* = 3 biological replicates per group). Two-tailed t-tests were used to calculate *P* values without adjustment.

Functionally, SLK phosphorylate polo-like kinase 1 (PLK1) at Thr210 (ref. 39). Our phosphoproteomic experiment revealed reduced PLK1 (T210) phosphorylation in CoCl_2_-treated cells (Extended Data Fig. 9a), while quantitative proteomics showed no changes in SLK or PLK1 protein abundance (Supplementary Fig. 4a,b), indicating decreased kinase activity rather than altered expression. In vitro kinase assays using His-tagged SLK purified from CoCl_2_-treated versus untreated H293T cells confirmed reduced PLK1 (T210) phosphorylation (Extended Data Fig. 9b). Semi-quantitative estimation of 2HG modification occupancy further revealed that CoCl_2_ treatment increased 2HG modification at SLK (S719) in both endogenous and overexpressed systems (Extended Data Fig. 9c,d).

**Extended Data Fig. 10 |:**
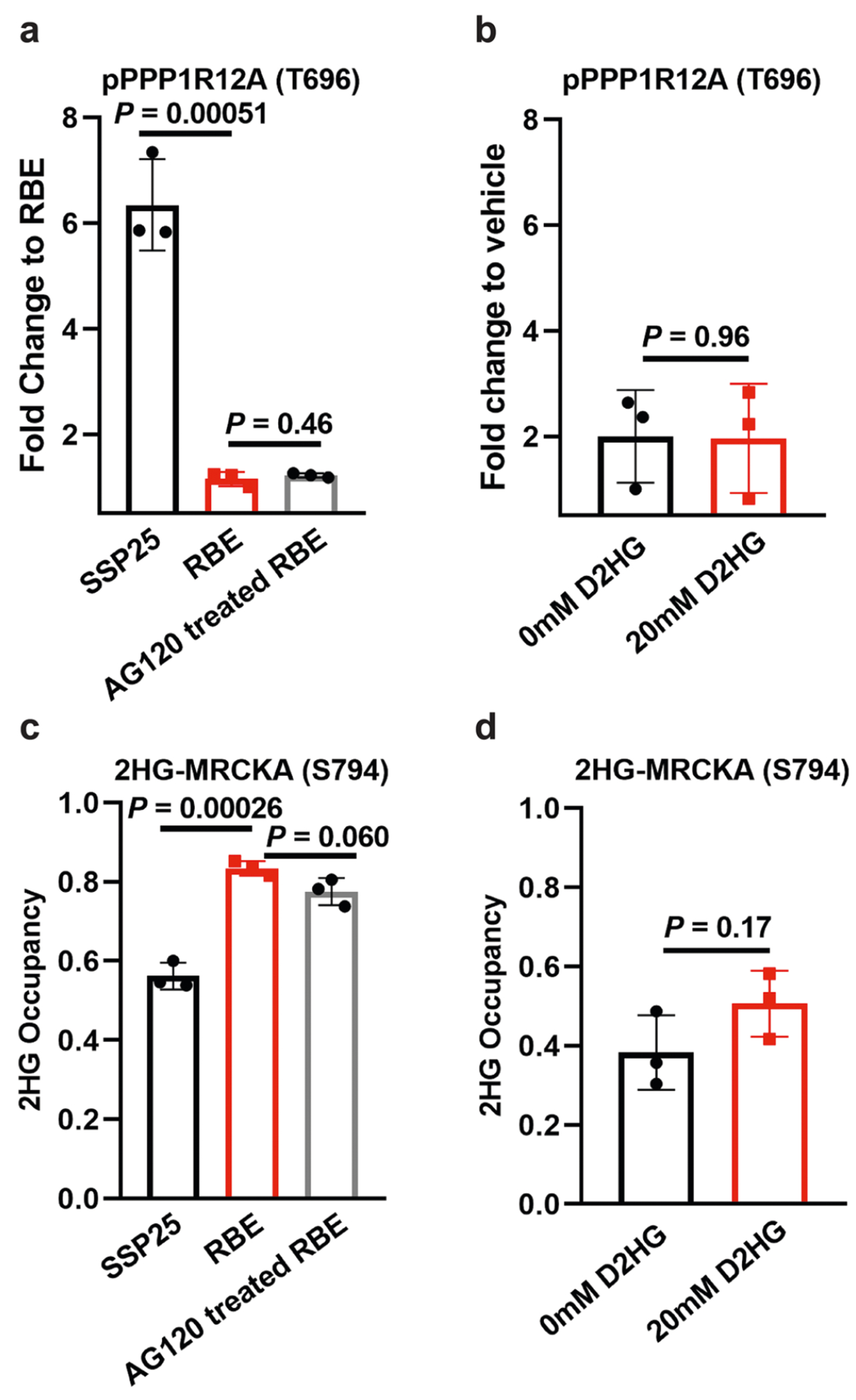
Quantitative analysis of D2HG modification and MRCKA activity. (**a**) MS^1^ TIC-normalized peak area fold changes of phosphorylated PPP1R12A (T696) in ICC cells with different genotypes: wild-type SSP25, IDH1-mutant RBE, and AG120-treated RBE, relative to RBE; (**b**) MS^1^ TIC-normalized peak area fold changes of phosphorylated PPP1R12A (T696) in the in-vitro kinase assay system, relative to vehicle control. (**c**) 2HG occupancy of MRCKA in ICC cells with different genotypes: wild-type SSP25, IDH1-mutant RBE, and AG120-treated RBE. (**d**) 2HG occupancy of MRCKA in purified His-MRCKA. In (**c,d**), values were calculated by comparing the peak areas of modified and unmodified MRCKA (S794) detected in the same LC–MS run, using the formula: *f*_mod_ = *A*_mod_/(*A*_mod_ + *A*_unmod_). Data represent mean ± s.d. (*n* = 3 biological replicates per group). Two-tailed t-tests were used to calculate *P* values without adjustment.

A similar effect was observed for myotonic dystrophy kinase-related CDC42-binding kinase alpha (MRCKA), a downstream effector of the small GTPase CDC42 and a therapeutic target owing to its role in tumour growth and metastasis^40^. D2HG modification at S794 increased on exogenous D2HG treatment in H293 cells, and correlated with endogenous D2HG levels across ICC cell lines of different genotypes (Figs. 1d, right, and 4d). Site assignment was validated with synthetic isotopically labelled peptides (Fig. 4e,f). MRCKA is known to phosphorylate PPP1R12A at T696 (ref. 41), and we observed decreased PPP1R12A (T696) phosphorylation in IDH1-mutant RBE cells relative to wild-type SSP25 cells in our quantitative phosphoproteomics experiment (Extended Data Fig. 10a). Protein abundances of MRCKA and PPP1R12A were unchanged (Supplementary Fig. 4c,d), indicating direct inhibition by D2HG modification. This observation aligns with previous reports linking reduced MRCKA activity to slower cell growth^42^ and may help explain the attenuated proliferation of IDH mutant cells^43^. To directly assess functional impact, we expressed MRCKA in H293T cells with or without 20 mM D2HG treatment. In vitro kinase assays revealed a slight change in PPP1R12A (T696) phosphorylation (Extended Data Fig. 10b). Occupancy analysis showed that although D2HG modification increased with treatment, overall 2HG stoichiometry remained markedly lower than in IDH1-mutant RBE cells (Extended Data Fig. 10c,d). This weaker inhibition probably reflects two factors, dilution of modification owing to MRCKA overexpression and reduced efficiency of exogenous D2HG incorporation compared with endogenous production.

To explore the mechanisms underlying reduced kinase activity with 2HG modifications, we modelled MRCKA and SLK structures using AlphaFold 3. Mimicking acidic 2HG modification via mutagenesis strategy^44^ revealed that 2HG modification of SLK (S719) created a barrier at the active site pocket, potentially hindering substrate access (Fig. 4g). Free energy changes (ΔΔ*G* = −0.56 kcal mol^−1^) further supported SLK destabilization upon S719 modification. Similarly, the dimerization of MRCKA, essential for activation^45^, was destabilized. The modification (S794), located at the coiled-coil domain responsible for dimer formation (Fig. 4h), probably induced electrostatic repulsion, reducing kinase activity. Free energy calculations supported this hypothesis (ΔΔ*G* = −0.93 kcal mol^−1^).

## Discussion

As IDH1 and IDH2 are the most frequently mutated metabolic genes in human cancer, intense efforts have been made to study their biochemical mechanisms and clinical implications^3^. A majority of studies have focused on D2HG functioning as a competitor of α-KG to cause epigenetic alteration or to antagonize other α-KG-dependent enzymes^9^. We report here the discovery and validation of the covalent modification by D2HG, *O*-2-hydroxyglutarylation. To facilitate the sensitive detection of this modification, we developed a chemical proteomic approach employing sequential trypsin-GluC digestion and polyMAC enrichment. These covalent modifications were confirmed through exogenous treatment, isotopic metabolic flux analysis, neutral loss in tandem mass spectrometry and synthetic peptide authentication.

Chirality is a fundamental characteristic of biological systems, as biomolecules such as nucleic acids and proteins are composed of stereospecific building blocks. Enantiomer-specific PTMs have recently emerged as a new layer of regulation. For instance, D- and L-lactylation were independently identified and shown to engage distinct cellular pathways^46^. In this context, our identification of covalent protein modifications driven by both D2HG and L2HG further underscores the role of metabolite chirality in shaping protein function and signalling. Although both arise from α-KG, their production is context-dependent: D2HG is predominantly generated by mutated IDH enzymes, whereas L2HG accumulates via promiscuous enzymatic activity under metabolic stress (acidosis or hypoxia)^11^. These enantiomers therefore exert distinct effects on cellular fate—shaping cancer progression, metabolic adaptation and immune regulation through unique pathways. Differences in source, function and target affinity between D2HG and L2HG are consistent with our finding that they modify distinct protein targets. Our findings provide a framework for exploring chirality dependent protein modifications—specifically *O*-2-hydroxyglutarylation in cancer biology—providing opportunities for therapeutic intervention.

The observation of crosstalk between D/L-2HG modification and phosphorylation is also quite interesting. Specifically, we identified D2HG-modified MRCKA and L2HG-modified SLK exhibited reduced kinase activity, with modification levels correlating to endogenous D2HG and L2HG concentrations, respectively. The discovery of D2HG-modified MRCKA (cdc42bka) establishes a curious connection with a previous study in which D2HG was reported to disrupt the association between the small GTPase Cdc42 and its interacting kinase, leading to the suppression of c-jun N-terminal kinase activation and apoptosis^47^. Interestingly, MRCKA is a known Cdc42-binding kinase and an important downstream effector^42^. Our results suggest that the D2HG modification on MRCKA inhibit the kinase activity, contributing to tumorigenesis driven by mutant IDH1. However, further biochemical experiments are needed to validate this inhibition mechanism.

Given the central role of D2HG and L2HG in cancer, exploring the impact of their modifications in various cancer contexts will be crucial. Two limits should be noted in our study. First, we cannot definitively assign the stereochemistry of each endogenous PTM. We infer D versus L from dose/time trends and from links to endogenous metabolite levels, but this is indirect and needs direct structural proof. Second, polyMAC has limited selectivity, increasing false positives and complicating site localization when MS/MS b/y-ion coverage is incomplete; isobaric modifications may also slip in. To address these gaps, we need enantioselective enrichment tools (antibodies or chemical probes) and structural methods (for example, NMR, cryo-EM). Additionally, beyond *O*-linkages, 2HG may also modify Lys, Cys, Asp or Glu, which should be tested in future work.

From a chemical standpoint, 2HG may modify S/T/Y residues through either of its two carboxyl groups. Previous studies of α-hydroxy acids indicate a preference for esterification at the α-carboxyl group, driven by intramolecular hydrogen bonding that enhances carbonyl electrophilicity and promotes nucleophilic attack^48,49^. This chemistry suggests that α-carboxyl ester formation may be favoured for 2HG; however, the precise esterification site and underlying reaction mechanism remain unresolved. Definitive assignment will require more advanced analytical methods capable of detailed structural characterization at the modification site.

Mechanistically, the stereoselective preference observed suggests that *O*-2-hydroxyglutarylation is at least partially enzyme catalysed, analogous to *O*-acylations, such as palmitoylation^50^. Although non-enzymatic pathways involving reactive 2HG-CoA intermediates remain possible—and D2HG-CoA has been reported in prokaryotes^51^—we did not detect such intermediates in mammalian cells (Supplementary Fig. 5), suggesting that this is unlikely to be the predominant route. Understanding whether these modifications are dynamically regulated by specific ‘writers’ and ‘erasers’ and identifying their ‘readers’ remains an important avenue for future research.

## Online content

Any methods, additional references, Nature Portfolio reporting summaries, source data, extended data, supplementary information, acknowledgements, peer review information; details of author contributions and competing interests; and statements of data and code availability are available at https://doi.org/10.1038/s41557-026-02093-x.

## Methods

### Reagents

Sequencing grade trypsin and GluC were obtained from Thermo Fisher and Sigma, respectively. PolyMAC was obtained from Tymora. Isotopic labelled d_4_ sodium (*RS*)-2-hydroxyglutarate (2,3,3-D_3_; optical density, 98%) was purchased from Cambridge Isotope Laboratories, which was called as d_4_-D/L-2HG hereafter. Other reagents were obtained from Sigma unless otherwise noted.

### Cell culture and treatment

ICC cell lines with different genotypes (RBE and SSP25) were provided by N. Bardeesy (Massachusetts General Hospital). H293T cells were obtained from American Type Culture Collection (catalogue no. CRL-3216). All cells were cultured at 37 °C with 5% CO_2_. ICC cells were cultured in RPMI medium (Thermo Fisher Scientific). H293T cells were cultured in Dulbecco’s modified Eagle medium (Gibco). All media were supplemented with 10% fetal bovine serum (FBS, Thermo Fisher Scientific), 100 U ml^−1^ penicillin and 100 U ml^−1^ streptomycin (Thermo Fisher Scientific).

For hypoxic conditions, cells were treated with varying concentrations of the hypoxia inducer CoCl_2_ (0 μM, 100 μM, 200 μM and 500 μM) for 24 hours or grown in a specialized, humidified chamber equilibrated with 1% oxygen, 94% nitrogen and 5% carbon dioxide for the indicated duration.

For isotopic metabolic flux experiments, cells were washed once with warm PBS and replenished with fresh RPMI with 10% FBS before labelling by d_4_-D/L-2HG. The d_4_-D/L-2HG (25 mM) was added into the media and cells were collected after 24 h.

### Cell collection and lysis

Cells were collected when reaching about 80% confluence. Cells were washed with cold PBS two times and scraped from the dish and transferred to a microcentrifuge tube. After that, cells were resuspended in lysis buffer (8 M urea in 50 mM Tris–HCl buffer, pH 8.0) with one tablet of EDTA-free protease inhibitor Cocktail, and then sonicated on ice for three 1-min rounds at 15% amplitude. The lysates were then centrifuged at 12,000*g*, at 4 °C for 20 min. The supernatant was transferred to a new tube. Total protein concentrations were determined by bicinchoninic acid protein assay kit (Thermo Scientific Pierce).

### Trypsin/GluC sequential digestion

A 1 M dithiothreitol (DTT) stock solution was added into cell lysates to a final concentration of 10 mM. The obtained solution was vortexed and incubated at 30 °C for 45 min. Then, 0.6 M chloroacetamide stock solution was added to a final concentration of 30 mM, followed by vortexing and incubation at room temperature in the dark for 30 min. Afterwards, another 1 M DTT was added to a final concentration of 10 mM, followed by vortexing and incubation at room temperature for 10 min. The obtained proteins were digested by a filter-aided sample preparation method (FASP). In general, the protein solution was transferred into a 10-kDa FASP filter and buffer-exchanged to 50 mM ammonium bicarbonate. Trypsin (enzyme:sample = 1:50) was added into the solution and incubated at 37 °C for 16 h. Then, the same volume of phosphate buffer (pH 7.5) and GluC (enzyme:sample = 1:50) was added into the solution and incubated at 37 °C for 8 h. Peptides were eluted from the membrane by centrifuging for 15 min at 12,000*g*. A 100-μl portion of water was used for a second elution. The peptides were desalted with C18 spin columns. The obtained solution was lyophilized for further use.

### Enrichment of 2HG-modified peptides by polyMAC

The 2HG peptides were enriched using polyMAC beads. First, 50 μl of polyMAC beads (Tymora) were rotated for 1 min and quickly spun down. The supernatants were removed, and 200 μl of loading buffer (0.01% trifluoroacetic acid (TFA), 20 mM glycolic acid (GA), 50% acetonitrile (ACN)) was added to the tubes, followed by rotation, centrifugation and removal of supernatants.

Next, 500 μl of loading buffer was added to the polyMAC, and the beads were split into three tubes (tube I: 200 μl; tube II: 200 μl; tube III: 100 μl). Enrichment was then performed using the sequential enrichment method.

Then, 100 μg of peptides dissolved in 500 μl of loading buffer was added to tube I, followed by incubation at room temperature with shaking for 30 min. The polyMAC beads were then spun at 2,000*g* for 1 min, and the supernatant was transferred to tube II. This was incubated at room temperature with shaking for 20 min, followed by spinning at 2,000*g* for 1 min and the supernatant transferred to tube III. Finally, the mixture was incubated at room temperature with shaking for 10 min.

After that, the polyMAC beads in tube III were spun down at 2,000*g* for 1 min and the supernatants were discarded. The three tubes of polyMAC were combined by using 200 μl of washing buffer A (0.1% TFA in 50% ACN), followed by spinning down at 2,000*g* for 1 min and discard of the supernatants. The polyMAC beads were then washed with 200 μl of washing buffer A (0.1% TFA in 50% ACN) four times and 200 μl of washing buffer B (80% ACN) one time. After centrifugation at 2,000*g* for 1 min and discarding the supernatants, the peptides were eluted from polyMAC beads with 150 μl of elution buffer (400 mM NH_4_OH in 50% ACN) by shaking at 1,100 rpm for 30 min at room temperature. The elution step was repeated once. The obtained eluted solution was collected and dried down in a SpeedVac and stored at −80 °C.

### Phosphopeptide enrichment

The phosphopeptides were enriched using polyMAC beads. First, 100 μg dried tryptic peptides were dissolved into 200 μl of loading buffer (100 mM GA, 1% TFA, 50% ACN) in a non-stick microfuge tube. Then, 50 μl of polyMAC reagent was added and shaken for 15 min. After that, the mixture was pipetted up and down a few times and the whole solution transferred to the spin column and spun down at 100*g* for 30 seconds to remove the unbonded peptides. Then, the polyMAC beads were sequentially washed with loading buffer, washing buffer I (25 mM GA, 0.2% TFA, 80% ACN) and washing buffer II (80% ACN) three times. Finally, the attached phosphopeptides were eluted from the polyMAC beads using 50 μl elution buffer (400 mM NH_4_OH, 50% ACN) two times, followed by drying of the samples in a SpeedVac.

### Expression and purification of target proteins in H293T cells

Plasmids encoding C-terminal His-tagged human SLK (pCMV3-His-SLK), MRCKA (pCMV3-His-MRCKA) and PPP1R12A (pCMV3-His-PPP1R12A), as well as recombinant His-tagged human PLK1 protein, were purchased from Sino Biological. H293T cells were transfected with these plasmids using Lipofectamine 3000 (Thermo Fisher) following the manufacturer’s protocol. For treatment groups, cells were incubated with CoCl_2_ or D2HG 24 h after transfection and cultured for an additional 24 h before collection. Untreated controls were cultured for the same duration. Non-transfected cells were also collected as negative control.

Cell pellets were resuspended in 8 ml lysis buffer (25 mM Tris–HCl pH 8.0, 200 mM NaCl, 5 mM imidazole and Pierce protease/phosphatase inhibitor). Cells were lysed by sonication on ice, and soluble fractions were collected by centrifugation (12,000*g*, 20 min, 4 °C). Supernatants were loaded onto a 2 ml Ni–NTA column (Qiagen), washed with 20 ml wash buffer (100 mM Tris–HCl pH 8.0, 1.5 M NaCl, 50 mM imidazole) and eluted with 10 ml elution buffer (25 mM Tris HCl pH 8.0, 200 mM NaCl, 200 mM imidazole). Eluates were concentrated and buffer-exchanged into 50 mM Tris–HCl (pH 7.4) using 50-kDa Amicon Ultra centrifugal filters.

### In vitro kinase assay and protein digestion for substrate phosphorylation profiling

Purified kinases (SLK or MRCKA, 100 nM) were incubated with their respective substrates (PLK1 or PPP1R12A, 200 nM) in kinase buffer (50 mM Tris–HCl, pH 7.4). Reactions were initiated by addition of 10× ATP/MgCl_2_/MnCl_2_ buffer to a final concentration of 10 mM MgCl_2_, 1 mM MnCl_2_ and 500 μM ATP, followed by incubation at 37 °C for 3 h. Reaction mixtures were buffer-exchanged into 50 mM ammonium bicarbonate (pH 8.0) using 50-kDa Amicon Ultra centrifugal filters.

Proteins were reduced with DTT (10 mM final concentration) at 30 °C for 2 h, then alkylated with chloroacetamide (30 mM final concentration) at room temperature in the dark for 1 h. Samples were digested with trypsin at a 1:50 enzyme-to-protein ratio and incubated at 37 °C for 16 h. Peptides were recovered by centrifugation (12,000*g*, 15 min), washed once with 200 μl water and re-centrifuged under the same conditions. Filtrates were combined, dried in a SpeedVac and analysed by LC–MS/MS in Parallel reaction monitoring (PRM)–parallel accumulation-serial fragmentation (PASEF) mode for substrate phosphorylation profiling.

### Proteomics LC–MS analysis

Samples were measured on a TIMS-TOF HT (Bruker Daltonics) with a reversed phase Evosep One (Evosep). For peptide separation, we used the standard SPD methods. The mass spectrometer was operated in a data-dependent acquisition PASEF mode with ten PASEF scans per topN acquisition cycle. The accumulation and ramp time for the dual TIMS analyser were set to 100 ms as a duty cycle. Full MS data were acquired in a mass range of 100–1,700 *m*/*z* and an inverse reduced mobility (1/*K0*) range of 0.6–1.6 V s^−1^ cm^−2^. The collision energy was ramped as a function of increasing mobility starting from 20 eV at 0.6 V s^−1^ cm^−2^ to 60 eV at 1.6 V s^−1^ cm^−2^. Representative 2HG-modified peptides were validated by PRM–PASEF. Transition lists were generated from DDA results and included retention time ranges, precursor *m*/*z* values, charge states and ion mobility ranges for each peptide.

### Reporting summary

Further information on research design is available in the Nature Portfolio Reporting Summary linked to this article.

## Extended Data

## Supplementary Material

EFig1

Fig3Data

Efig4

Efig3

Efig5

Efig6

Efig7

SuppD3

SuppD1

SuppD2

SuppD4

RptSum

SuppTbl

Supp

Fig4Data

Fig1Data

Fig2Data

Efig2

Efig8

Efig9

Efig10

The online version contains supplementary material available at https://doi.org/10.1038/s41557-026-02093-x.

## Data Availability

The raw mass spectrometry proteomics data, protein identification and quantification results have been deposited with the ProteomeXchange Consortium via the PRIDE partner repository with the dataset identifier PXD072530. The Supplementary Tables are published alongside this paper. The reviewed human fasta database was downloaded from UniProt in March 2024 (https://www.uniprot.org/uniprotkb). Source data are provided with this paper.

## References

[1] Liu Y & Yang CZ Oncometabolites in cancer: current understanding and challenges. Cancer Res. 81, 2820–2823 (2021). 33762356 10.1158/0008-5472.CAN-20-3730

[2] Sulkowski PL Oncometabolites suppress DNA repair by disrupting local chromatin signalling. Nature 582, 586–591 (2020). 32494005 10.1038/s41586-020-2363-0 PMC7319896

[3] Pirozzi CJ & Yan H The implications of IDH mutations for cancer development and therapy. Nat. Rev. Clin. Oncol. 18, 645–661 (2021). 34131315 10.1038/s41571-021-00521-0

[4] Liu ACH Targeting STAT5 signaling overcomes resistance to IDH inhibitors in acute myeloid leukemia through suppression of stemness. Cancer Res. 82, 4325–4339 (2022). 36150062 10.1158/0008-5472.CAN-22-1293

[5] Thomas D Dysregulated lipid synthesis by oncogenic IDH1 mutation is a targetable synthetic lethal vulnerability. Cancer Discov. 13, 496–515 (2023). 36355448 10.1158/2159-8290.CD-21-0218 PMC9900324

[6] Farshidfar F Integrative genomic analysis of cholangiocarcinoma identifies distinct IDH mutant molecular profiles. Cell Rep. 18, 2780–2794 (2017). 28297679 10.1016/j.celrep.2017.02.033 PMC5493145

[7] Wu MJ Mutant IDH inhibits IFNγ-TET2 signaling to promote immunoevasion and tumor maintenance in cholangiocarcinoma. Cancer Discov. 12, 812–835 (2022). 34848557 10.1158/2159-8290.CD-21-1077 PMC8904298

[8] Yen KE, Bittinger MA, Su SM & Fantin VR Cancer-associated IDH mutations: biomarker and therapeutic opportunities. Oncogene 29, 6409–6417 (2010). 20972461 10.1038/onc.2010.444

[9] Han S IDH mutation in glioma: molecular mechanisms and potential therapeutic targets. Br. J. Cancer 122, 1580–1589 (2020). 32291392 10.1038/s41416-020-0814-x PMC7250901

[10] Losman JA, Koivunen P & Kaelin WG 2-Oxoglutarate-dependent dioxygenases in cancer. Nat. Rev. Cancer 20, 710–726 (2020). 33087883 10.1038/s41568-020-00303-3

[11] Ye D, Guan KL & Xiong Y Metabolism, activity, and targeting of D- and L-2-hydroxyglutarates. Trends Cancer 4, 151–165 (2018). 29458964 10.1016/j.trecan.2017.12.005 PMC5884165

[12] Koivunen P Transformation by the *(R)*-enantiomer of 2-hydroxyglutarate linked to EGLN activation. Nature 483, 484–488 (2012). 22343896 10.1038/nature10898 PMC3656605

[13] Carbonneau M The oncometabolite 2-hydroxyglutarate activates the mTOR signalling pathway. Nat. Commun 7, 12700 (2016). 27624942 10.1038/ncomms12700 PMC5027283

[14] Figlia G, Willnow P & Teleman AA Metabolites regulate cell signaling and growth via covalent modification of proteins. Dev. Cell 54, 156–170 (2020). 32693055 10.1016/j.devcel.2020.06.036

[15] Liu DY Discovery of itaconate-mediated lysine acylation. J. Am. Chem. Soc 145, 12673–12681 (2023). 37271942 10.1021/jacs.3c02332

[16] Li XL, Gluth A, Zhang T & Qian WJ Thiol redox proteomics: characterization of thiol-based post-translational modifications. Proteomics 23, e2200194 (2023). 37248656 10.1002/pmic.202200194 PMC10764013

[17] Geiszler DJ PTM-shepherd: analysis and summarization of post-translational and chemical modifications from open search results. Mol. Cell. Proteomics 20, 100018 (2021). 33568339 10.1074/mcp.TIR120.002216 PMC7950090

[18] Ahrné E, Müller M & Lisacek F Unrestricted identification of modified proteins using MS/MS. Proteomics 10, 671–686 (2010). 20029840 10.1002/pmic.200900502

[19] Rykær M, Svensson B, Davies MJ & Hägglund P Unrestricted mass spectrometric data analysis for identification, localization, and quantification of oxidative protein modifications. J. Proteome Res 16, 3978–3988 (2017). 28920440 10.1021/acs.jproteome.7b00330

[20] Intlekofer AM Hypoxia induces production of L-2-hydroxyglutarate. Cell Metab. 22, 304–311 (2015). 26212717 10.1016/j.cmet.2015.06.023 PMC4527873

[21] Oldham WM, Clish CB, Yang Y & Loscalzo J Hypoxia-mediated increases in L-2-hydroxyglutarate coordinate the metabolic response to reductive stress. Cell Metab. 22, 291–303 (2015). 26212716 10.1016/j.cmet.2015.06.021 PMC4526408

[22] Wu MJ Mutant IDH1 inhibition induces dsDNA sensing to activate tumor immunity. Science 385, eadl6173 (2024). 38991060 10.1126/science.adl6173 PMC11602233

[23] Iliuk AB, Martin VA, Alicie BM, Geahlen RL & Tao WA In-depth analyses of kinase-dependent tyrosine phosphoproteomes based on metal ion-functionalized soluble nanopolymers. Mol. Cell. Proteomics 9, 2162–2172 (2010). 20562096 10.1074/mcp.M110.000091 PMC2953913

[24] Li YZ Functional profiling of serine, threonine and tyrosine sites. Nat. Chem. Biol 21, 532–543 (2024). 39313591 10.1038/s41589-024-01731-0

[25] Wang P Oncometabolite D-2-hydroxyglutarate Inhibits ALKBH DNA repair enzymes and sensitizes IDH mutant cells to alkylating agents. Cell Rep. 13, 2353–2361 (2015). 26686626 10.1016/j.celrep.2015.11.029 PMC4694633

[26] Golub D Mutant isocitrate dehydrogenase inhibitors as targeted cancer therapeutics. Front. Oncol 9, 00417 (2019). 10.3389/fonc.2019.00417 PMC6534082 31165048

[27] Wang QX Challenges for the development of mutant isocitrate dehydrogenases 1 inhibitors to treat glioma. Eur. J. Med. Chem 257, 115464 (2023). 37235998 10.1016/j.ejmech.2023.115464

[28] Chakraborty A, Bose R & Bose K Unraveling the dichotomy of enigmatic serine protease HtrA2. Front. Mol. Biosci 9, 824846 (2022). 35187085 10.3389/fmolb.2022.824846 PMC8850690

[29] Wong HSC & Chang WC Single-cell melanoma transcriptomes depicting functional versatility and clinical implications of STIM1 in the tumor microenvironment. Theranostics 11, 5092–5106 (2021). 33859736 10.7150/thno.54134 PMC8039943

[30] Agrawal-Singh S HOXA9 forms a repressive complex with nuclear matrix-associated protein SAFB to maintain acute myeloid leukemia. Blood 141, 1737–1754 (2023). 36577137 10.1182/blood.2022016528 PMC10113176

[31] Fan CY, Deng Q & Zhu TF Bioorthogonal information storage in L-DNA with a high-fidelity mirror-image Pfu DNA polymerase. Nat. Biotechnol 39, 1548–1555 (2021). 34326549 10.1038/s41587-021-00969-6

[32] Wang YJ Expedited mapping of the ligandable proteome using fully functionalized enantiomeric probe pairs. Nat. Chem 11, 1113–1123 (2019). 31659311 10.1038/s41557-019-0351-5 PMC6874898

[33] Joyce AW & Searle BC Computational approaches to identify sites of phosphorylation. Proteomics 24, e2300088 (2024). 37897210 10.1002/pmic.202300088 PMC12188548

[34] Bashyal A & Brodbelt JS Uncommon posttranslational modifications in proteomics: ADP-ribosylation, tyrosine nitration, and tyrosine sulfation. Mass Spectrom. Rev 43, 289–326 (2024). 36165040 10.1002/mas.21811 PMC10040477

[35] Remus D Concerted loading of Mcm2–7 double hexamers around DNA during DNA replication origin licensing. Cell 139, 719–730 (2009). 19896182 10.1016/j.cell.2009.10.015 PMC2804858

[36] Hanssen-Bauer A, Solvang-Garten K, Akbari M & Otterlei M X-ray repair cross complementing protein 1 in base excision repair. Int. J.Mol. Sci 13, 17210–17229 (2012). 23247283 10.3390/ijms131217210 PMC3546746

[37] Li KJ A peptide-centric local stability assay enables proteome-scale identification of the protein targets and binding regions of diverse ligands. Nat. Methods 22, 278–282 (2025). 39658593 10.1038/s41592-024-02553-7

[38] Ma SH *L2hgdh* deficiency accumulates L-2-hydroxyglutarate with progressive leukoencephalopathy and neurodegeneration. Mol. Cell. Biol 37, e00492–16 (2017). 28137912 10.1128/MCB.00492-16 PMC5376639

[39] Li J Polo-like kinase 1 regulates vimentin phosphorylation at Ser-56 and contraction in smooth muscle. J. Biol. Chem 291, 23693–23703 (2016). 27662907 10.1074/jbc.M116.749341 PMC5095422

[40] Leung T, Chen XQ, Tan I, Manser E & Lim L Myotonic dystrophy kinase-related Cdc42-binding kinase acts as a Cdc42 effector in promoting cytoskeletal reorganization. Mol. Cell. Biol 18, 130–140 (1998). 9418861 10.1128/mcb.18.1.130 PMC121465

[41] Wilkinson S, Paterson HF & Marshall CJ Cdc42-MRCK and Rho-ROCK signalling cooperate in myosin phosphorylation and cell invasion. Nat. Cell Biol 7, 255–261 (2005). 15723050 10.1038/ncb1230

[42] Kurimchak AM Functional proteomics interrogation of the kinome identifies MRCKA as a therapeutic target in high-grade serous ovarian carcinoma. Sci. Signal 13, eaax8238 (2020). 32071169 10.1126/scisignal.aax8238 PMC7294993

[43] Garrett M Metabolic characterization of isocitrate dehydrogenase (IDH) mutant and IDH wildtype gliomaspheres uncovers cell type-specific vulnerabilities. Cancer Metab. 6, 4 (2018). 29692895 10.1186/s40170-018-0177-4 PMC5905129

[44] Liang BW SARS-CoV-2 spike protein post translational modification landscape and its impact on protein structure and function via computational prediction. Research 6, 0078 (2023). 36930770 10.34133/research.0078 PMC10013967

[45] Tan I, Seow KT, Lim L & Leung T Intermolecular and intramolecular interactions regulate catalytic activity of myotonic dystrophy kinase-related Cdc42-binding kinase α. Mol. Cell. Biol 21, 2767–2778 (2001). 11283256 10.1128/MCB.21.8.2767-2778.2001 PMC86907

[46] Zhang D Lysine L-lactylation is the dominant lactylation isomer induced by glycolysis. Nat. Chem. Biol 21, 91–99 (2025). 39030363 10.1038/s41589-024-01680-8 PMC11666458

[47] Jiang B IDH1 mutation promotes tumorigenesis by inhibiting JNK activation and apoptosis induced by serum starvation. Cell Rep. 19, 389–400 (2017). 28402860 10.1016/j.celrep.2017.03.053

[48] Guerrero-Corella A, Fraile A & Alemán J Intramolecular hydrogen-bond activation: strategies, benefits, and influence in catalysis. ACS Org. Inorg. Au 2, 197–204 (2022). 35673681 10.1021/acsorginorgau.1c00053 PMC9164241

[49] Ansell RJ, Barrett SA, Meegan JE & Warriner SL On the interactions of alkyl 2-hydroxycarboxylic acids with alkoxysilanes: selective esterification of simple 2-hydroxycarboxylic acids. Chem. Eur. J 13, 4654–4664 (2007). 17443833 10.1002/chem.200601859

[50] Takada R Monounsaturated fatty acid modification of Wnt protein: its role in Wnt secretion. Dev. Cell 11, 791–801 (2006). 17141155 10.1016/j.devcel.2006.10.003

[51] Yu JL, Xia XX, Zhong JJ & Qian ZG A novel synthetic pathway for glutarate production in recombinant Escherichia coli. Process Biochem. 59, 167–171 (2017).

