# Author Correction: Discovery of chirally dependent protein modifications by D- and L-2-hydroxyglutarates

**DOI:** 10.1038/s41557-026-02145-2

**Published:** 2026-04-10

**Authors:** Zheng Zhang, Yi-Kai Liu, Zhuojun Luo, Meng-Ju Wu, Claudia N. Evans, Zihan Qu, Fanglei Xue, Zhijian Wang, Lia Stanciu, Zhong-Yin Zhang, Elizabeth I. Parkinson, Nabeel Bardeesy, W. Andy Tao

**Affiliations:** 1 Department of Biochemistry, Purdue University, West Lafayette, IN USA; 2 Cancer Center, Massachusetts General Hospital, Department of Medicine, Harvard Medical School, Boston, MA USA; 3 Broad Institute of Harvard and Massachusetts Institute of Technology, Cambridge, MA USA; 4 Division of Gastroenterology, Department of Medicine, University of Massachusetts Chan Medical School, Worcester, MA USA; 5 James Tarpo Jr. and Margaret Tarpo Department of Chemistry, Purdue University, West Lafayette, IN USA; 6 University of Technology Sydney, Sydney, New South Wales Australia; 7 School of Materials Engineering, Purdue University, West Lafayette, IN USA; 8 Borch Department of Medicinal Chemistry and Molecular Pharmacology, Purdue University, West Lafayette, IN USA; 9 Purdue Institute for Cancer Research, Purdue University, West Lafayette, IN USA

**Keywords:** Chemical modification, Proteomics

Correction to: *Nature Chemistry* 10.1038/s41557-026-02093-x, published online 17 March 2026.

In the version of this article initially published, there was an error in the spelling of Zhijian Wang’s first name (Zijian) which is now amended in the HTML and PDF versions of the article.

