# Supplementary material for: Discovery of chirally dependent protein modifications by D- and L-2-hydroxyglutarates
Source: Nat Chem. 2026 Mar 17;18(6):1033–41. doi: 10.1038/s41557-026-02093-x (PMC13193722; doi:10.1038/s41557-026-02093-x)
Supplement: Supplementary file 1 — Supplementary Figs. 1–5, Notes 1–5 and references. [file 41557_2026_2093_MOESM1_ESM.pdf]

nature chemistry

Supplementary information

https://doi.org/10.1038/s41557-026-02093-x

Discovery of chirally dependent protein modifications by D- and L-2-hydroxyglutarates

In the format provided by the authors and unedited

Table of Contents:

Supplementary Figs. 1-5	Page 2-6
Supplementary Note 1	Page 7
Supplementary Note 2	Page 8
Supplementary Note 3	Page 9
Supplementary Note 4	Page 10
Supplementary Note 5	Page 11-14
Supplementary Reference	Page 15

Supplementary Figures

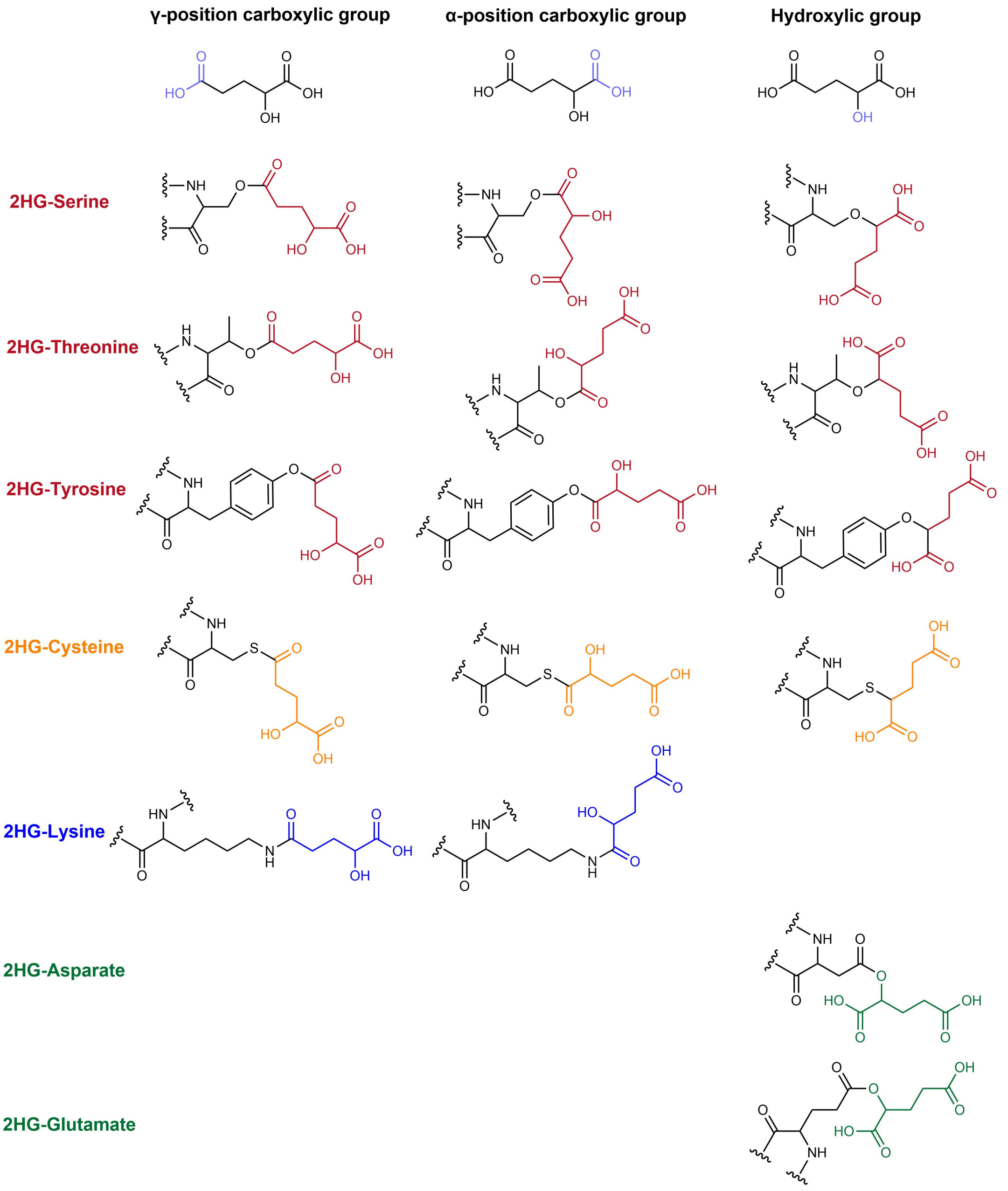

Supporting figure 1. Possible structure of 2HG modification.

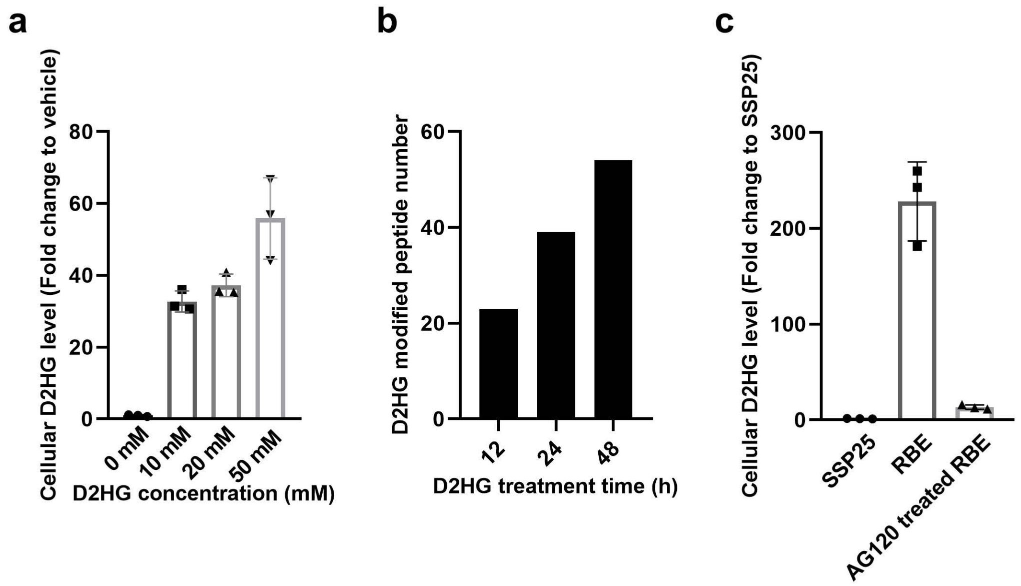

Supporting figure 2. Recognition of D2HG modified peptides. (**a**) Fold changes of endogenous D2HG in H293T cells treated with increasing concentrations of D2HG, relative to vehicle control. (**b**) Identification of D2HG-modified peptides via quantitative proteomics: H293T cells were treated with 20 mM D2HG over different time points. (**c**) Fold changes of endogenous D2HG in wild-type SSP25 cells, IDH1 mutant RBE cells, and AG120-treated RBE cells, relative to SSP25 cells. For quantitative proteomics (**b**), the peptides with a fold change > 1.2 and *P* value < 0.05 in the D2HG accumulated groups compared with the non-accumulated group were considered as D2HG modified peptides. Data represent mean ± s.d. (*n* = 3 biological replicates per group). Two-tailed t-tests were used to calculate *P* values without adjustment.

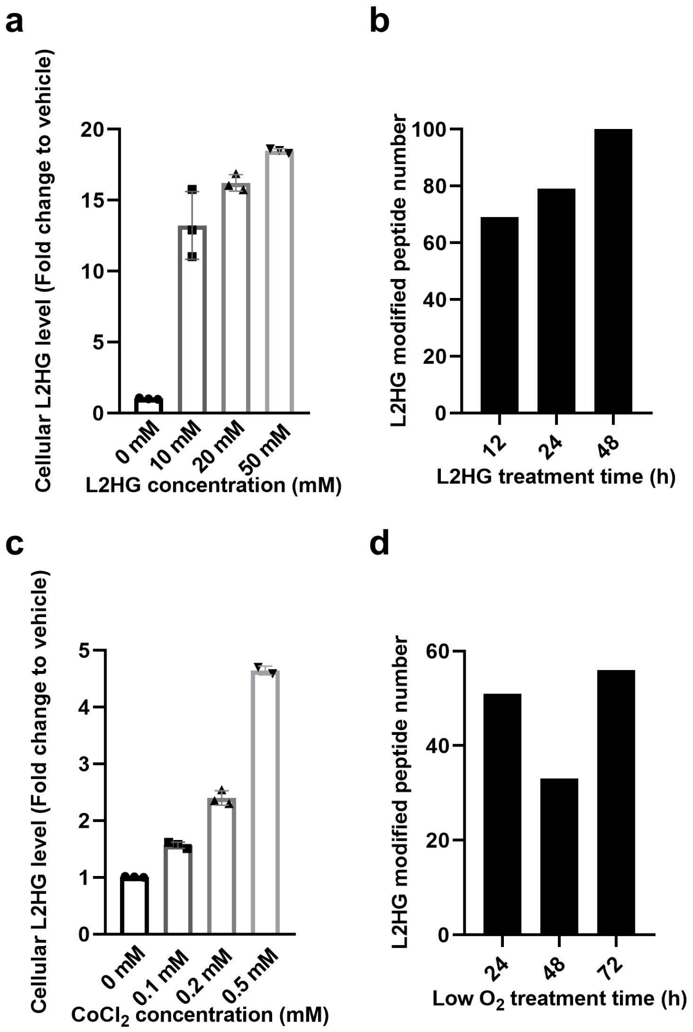

Supporting figure 3. Recognition of L2HG modified peptides. (**a,c**) Fold changes of endogenous L2HG in H293T cells treated with various concentrations of L2HG (**a**) and $CoCl_2$ (**c**), relative to vehicle control. (**b,d**) Identification of L2HG-modified peptides via quantitative proteomics: (**b**) H293T cells were treated with 20 mM L2HG over varying time points; (**d**) H293T cells were exposed to a low O_2 environment for different durations. For quantitative proteomics (**b, d**), the peptides with a fold change > 1.2 and *P* value < 0.05 in the L2HG accumulated groups compared with the non-accumulated group were considered as L2HG modified peptides. Data represent mean ± s.d. (*n* = 3 biological replicates per group). Two-tailed t-tests were used to calculate *P* values without adjustment.

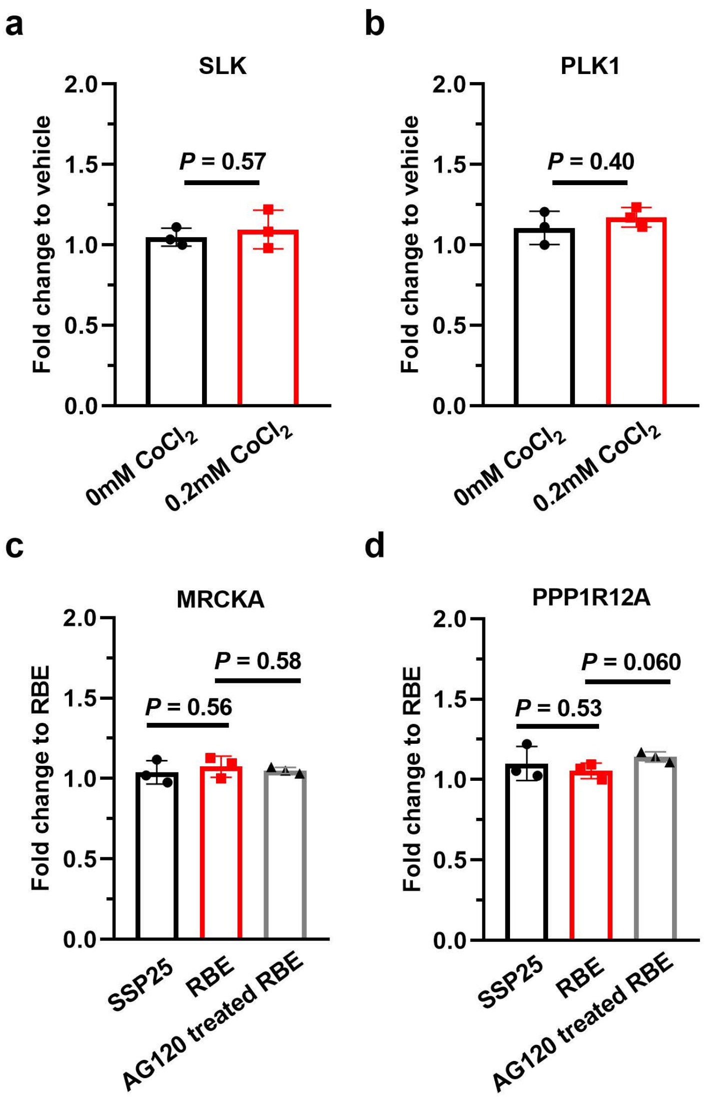

Supporting figure 4. Quantitative proteomic analysis of kinases and their substrates under treatment conditions. (**a**) Fold changes of SLK in H293 cells treat with 0 mM and 0.2 mM $CoCl_2$, relative to vehicle control. (**b**) Fold changes of PLK1 in H293 cells treat with 0 mM and 0.2 mM $CoCl_2$, relative to vehicle control. (**c**) Fold changes of MRCKA in wild-type SSP25 cells, IDH1-mutant RBE cells, and AG120-treated RBE cells,relative to RBE. (**d**) Fold changes of PPP1R12A in wild-type SSP25 cells, IDH1-mutant RBE cells, and AG120-treated RBE cells, relative to RBE. Data represent mean ± s.d. (n = 3 biological replicates per group). Two-tailed t-tests were used to calculate P values without adjustment.

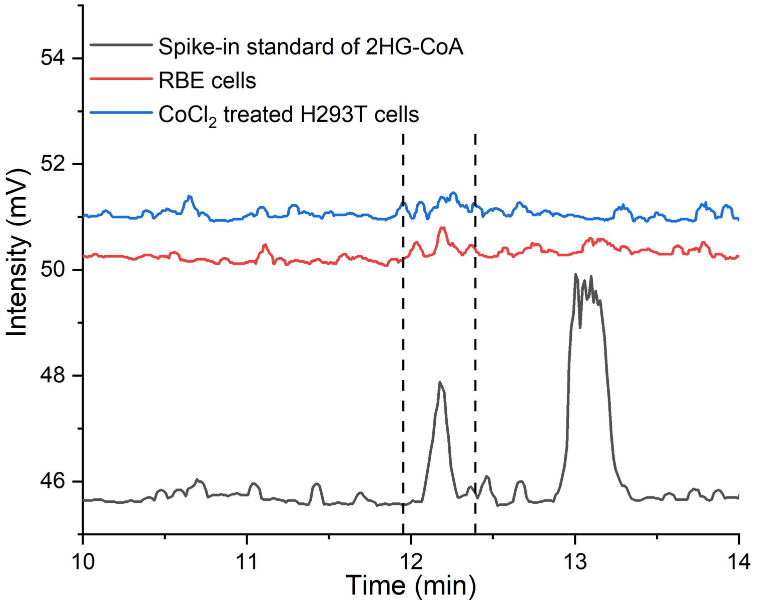

Supporting figure 5. LC-MS detection of 2HG–CoA. Targeted LC–MS analysis of 2HG–CoA was performed in multiple reaction monitoring (MRM) mode in IDH1-mutant RBE cells, $CoCl_2$-treated H293T cells, and cell lysates spiked with a synthetic 2HG–CoA standard. To enhance detectability, samples were derivatized by TMS-diazomethane methylation prior to LC–MS analysis, as described previously[1].

Supplementary Note 1. Optimization of polyMAC-based enrichment for 2HG modification.

We initially employed standard phosphopeptide protocols of polyMAC but yielded poor recovery of 2HG-modified peptides, likely due to competition from glycolic acid (25–100 mM) in the loading/wash buffers. By systematically optimizing buffer acidity and GA concentration, we reduced nonspecific binding of unmodified peptides and improved enrichment selectivity, resulting in ~2-fold 2HG-modified peptides compared with unenriched input (**Extended Data Fig. 2b, Table S2**).

We next evaluated protease choice, as tryptic peptides often contain abundant Asp and Glu residues whose carboxyl groups compete for polyMAC binding by Lewis acid-base interaction. GluC digestion (fewer acidic residues) improved detection relative to trypsin alone, and a sequential trypsin-GluC strategy gave the highest yield and selectivity for 2HG-modified peptides (**Extended Data Fig. 2b-c, Table S2**). Based on its superior identifications and specificity, we adopted the sequential trypsin-GluC workflow for subsequent experiments.

We then re-optimized enrichment for the sequential digestion strategy. Increasing buffer acidity and GA concentration enhanced identification of 2HG-modified peptides by protonating carboxyl groups and reducing interference from unmodified peptides, though excessive increases reduced recovery (**Extended Data Fig. 2e**). Under optimal conditions, recovery improved >10-fold over unenriched input, enabling identification of ~200 2HG modified peptides in RBE cells (**Table S3**). Reproducibility was high, with five technical replicates showing excellent correlation of peptide intensities (**Extended Data Fig. 2g**).

Supplementary Note 2. Proteomic data analysis

The raw files were processed using the FragPipe software 21.0, a robust software suite for DDA analysis. The data was searched against a reverse concatenated, nonredundant variant of the Human UniProt database (UniProt_Human_reviewed_26-03-2024.fasta) by the engine MSFragger (v4.0). A mass tolerance of 20 ppm was allowed for precursor ions and 20 ppm for fragment ions. Cysteine carbamidomethylation (+57.0215 Da) was chosen as static modification. 130.0266 Da (for 2HG modification) was set as variable modifications on serine, threonine and tyrosine. 15.9949 Da was set as variable modification on methionine. Trypsin, GluC or trypsin-GluC was set as the protease, allowing a maximum missed cleavage of three. The search results were statistically validated using Philosopher (v5.1.0), and PTMProphet was employed to compute PTM site localization probabilities. IonQuant (v1.10.12) was used for MS1 precursor intensity quantification, applying a minimum PTM site probability threshold of 0.75.

Peptide-spectrum matches (PSMs) were filtered to 1% FDR using a target-decoy database approach. Missing values were imputed using the minimum values in each dataset[2]. To identify high-confidence D- and L2HG-modified peptides, we applied a stepwise workflow: (1) peptides with fold change > 1.2 and $P < 0.05$ in D2HG or L2HG-accumulated groups were included in the whitelist, (2) while those with fold change < 0.833 and $P < 0.05$ were placed in the blacklist; (3) Blacklisted peptides were removed, and peptides identified only once were excluded. Two-tailed t-tests were used to calculate *P* values without adjustment.

MS/MS spectra of 2HG-modified peptides were manually inspected, requiring detection of at least two b/y ions containing the 2HG site. For quantification of 2HG modification and phosphorylation sites, we manually examined the extracted ion chromatograms (EICs) of modified peptides and integrated their peak areas using Bruker Compass DataAnalysis 6.1.

Supplementary Note 3. LC–MS detection of D/L-2HG in cells.

Metabolites were extracted from exponentially growing cells with 80% aqueous methanol (–80 °C) and analyzed by LC–MS. Total 2HG was quantified in negative mode using multiple reaction monitoring (MRM) on an Agilent 6460 QQQ LC–MS/MS. Separation was performed on a ZIC-HILIC column (3 μm, 2.0 × 100 mm, SeQuant®). Mobile phase A: 200 mM formic acid in LC–MS–grade water (pH adjusted to 3.25 with ammonium hydroxide); mobile phase B: acetonitrile. Chromatography was performed with isocratic elution (15% buffer A, 85% buffer B) for 30 min per sample. MRM transition for 2HG: 147-129. D/L-2HG standards were used as references for relative quantification of cellular 2HG.

This method does not resolve the enantiomers; it therefore reports total 2HG. However, their relative contributions can be inferred from biological context: D2HG accumulation is characteristic of IDH1-mutant cells, whereas L2HG is specifically elevated under hypoxia or $CoCl_2$ treatment. Thus, observed changes in total 2HG under these conditions largely reflect D2HG or L2HG, respectively.

Supplementary Note 4. Bioinformatic analysis and model construction

Functional annotation and statistical analyses

The 2HG modified proteins were subjected to GO functional annotation including molecule function and biological process by DAVID Bioinformatics Resources 6.8 (https://david.ncifcrf.gov/). For comparison between two groups, datasets were analyzed by a unpaired two-tailed Student's t-test without adjustment.

Prediction of 2HG modified protein models

MRCKA and SLK were selected as representative proteins for modelling with or without 2HG modifications. Protein structures were generated using AlphaFold 3[3]. Crystal structures of proteins were illustrated using Pymol 2.1.1. To mimic acidic 2HG modifications, serine residues at the modification sites were substituted with asparagine using conventional mutagenesis strategy[4, 5]. The thermodynamic stability of the proteins was assessed via changes in folding free energy (ΔΔG), calculated using SAAFEC-SEQ[6].

Supplementary Note 5. Compound Synthesis

Chemical synthesis of D/L Fmoc-Ser(2HG)-OH

(1) Synthesis of diethyl 2-hydroxypentanedioate (S1)

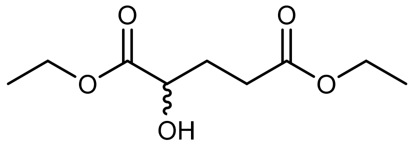

S1

1 eq. (1g, 5.208 mmol) of 2HG were weighted into a 250mL flask, followed by the addition of 40mL ethanol and 0.3eq. (0.4mL) of H_2SO4. The reaction was refluxed at 70°C overnight. The solvent was evaporated, followed by the addition of 20mL H_2O and neutralization using 1N KOH. The aqueous layer was extracted 3 times with 50mL of EtOAc and dried by Na_2SO4 and filtered. The filtrate was concentrated in vacuo. The crude was directly used for next step synthesis.

(2) Synthesis of diethyl 2-(benzyloxy) pentanedioate (S2)

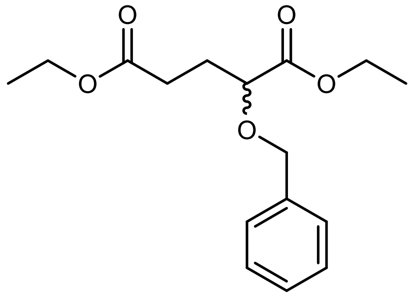

S2

Add dropwise a solution of Compound S1 (0.81g, 4mmol) in DMF (2mL) to a suspension of NaH (60% in mineral oil, 0.32g, 8mmol) in dry DMF (8mL) at 0°C. Stir the reaction for 30 min at 0°C. Add BnBr (0.953mL, 8mmol) to the mixture. Continue the stirring for 40 min. Stir the mixture at 0°C for 2h. Add TEA (1.13mL, 8mmol) and stirred for another 1h. Quench the reaction by addition of H_2O (50mL). Extract the reaction with Et_2O (5*8mL). Wash the extracts with brine and dry by Na_2SO4. Dry the solution and purify the product by column chromatography (petroleum ether : EtOAc = 10:1). MS (ESI): m/z calcd for $C_{16}H_{23}O_5^+$ $[M+H]^+$: 295.1; found: 295.1.

(3) Synthesis of 2-(benzyloxy) pentanedioic acid (S3)

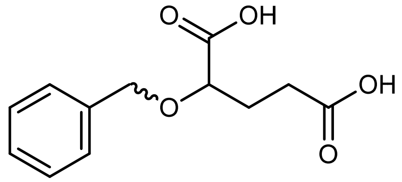

S3

To a solution of S2 (0.33g, 1.1224mmol) in 2.37mL EtOH at 0°C was added a solution of KOH (0.25g, 4.49mmol) in 2.37mL H_2O. The reaction was allowed to warm to RT and stirred overnight. After concentrating the solution in vacuo to remove EtOH, the remained aqueous phase was adjusted to pH 1-2 by HCl. Then dry the solution in vacuum. MS (ESI): m/z calcd for $C_{12}H_{13}O_5^-$ [M-H]$^-$: 237.1; found: 237.1.

(4) Synthesis of benzyl protected D/L-2HG (S4)

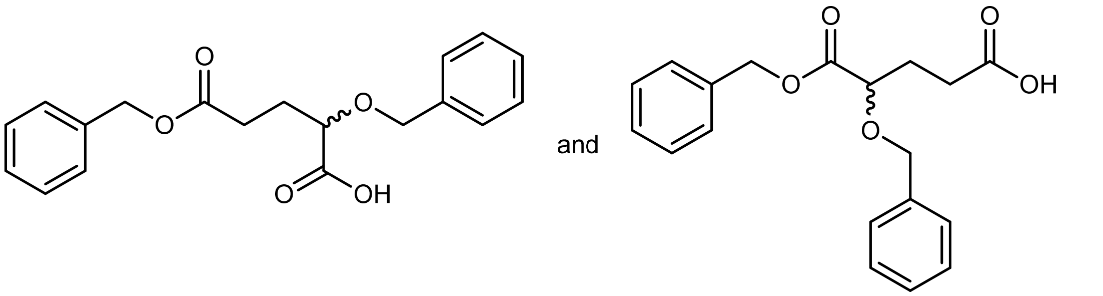

S4

Oxalyl chloride (1.36mmol, 0.17g, 116ul, 1.2eq) was added to a stirring solution of Compound S3 (0.27g, 1.1345mmol, 1.0eq) in DCM (1mL) at 0°C, followed by adding two drops of DMF. The reaction was allowed to react at RT for 3 h, followed by drying in vacuum. The crude was dissolved in 1mL DCM. The resulting solution was added dropwise to a solution of BnOH (1.3614mmol, 147mg, 1.2eq) and TEA (114.8mg, 1.1345mmol, 1eq) in 1mL THF at 0°C. The reaction was stirred for 1h at 0°C and for overnight at RT. The resulting solution was quenched by 0.1ml H_2O. After evaporation, the products were purified by C18-HPLC (5–90% MeOH over 20 min) to afford Compound S4. MS (ESI): m/z calcd for $C_{19}H_{19}O_5^-$ [M-H]$^-$: 327.1; found: 327.1.

(5) Synthesis of Fmoc-Ser(benzyl-2HG)-OH (S5)

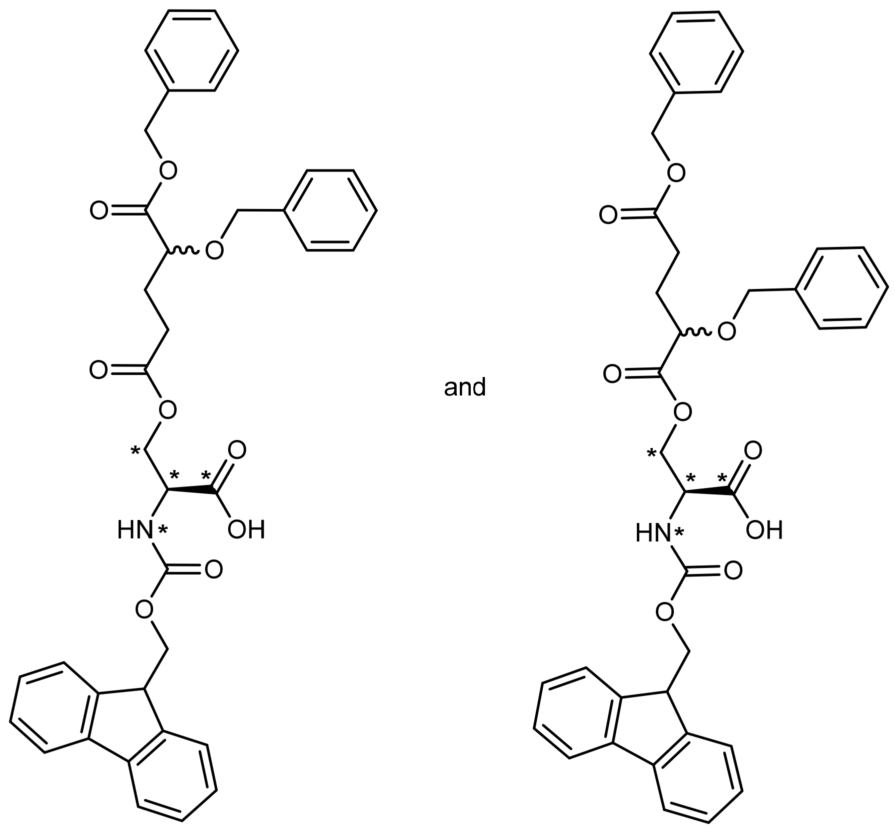

S5

Oxalyl chloride (87mg, 0.6860mmol, 1.5eq) was added to a stirring solution of Compound S4 (150mg, 0.4573mmol, 1.0eq) in DCM (1mL) at 0°C, followed by adding two drops of DMF. The reaction was allowed to react at RT for 4 h, followed by drying in vacuum. The crude was dissolved in 1mL DCM. The resulting solution was added dropwise to a solution of Fmoc-Ser($^{13}C_3$,^{15}N)-OH (224.3mg, 0.686mmol, 1.5eq) and TEA (92.55mg, 0.9146mmol, 2eq) in 1mL DCM at 0°C. The reaction was stirred for 1h at 0°C and for 1h at RT. The resulting solution was dried and the compound was purified by C18-HPLC (5%-90% MeOH in 30 min). S5: HRMS (ESI): m/z calcd for $C_{37}H_{36}NO_9^+$($^{13}C_3$, ^{15}N) $[M+H]^+$: 642.246; found: 642.243. 1H-NMR (300 MHz, $CDCl_3$) δ 7.75 (m, 2H), 7.60 (t, 2H), 7.39 (m, 2H), 7.24-7.42 (m, 12H), 5.8 (d, 1H), 5.05 (d, 2H), 4.64 (d, 2H), 4.53 (d, 1H), 4.38 (m, 3H), 4.22 (t, 1H), 4.02 (m, 1H), 3.48 (s, 2H), 3.23 (br, 1H), 2.43 (m, 1H), 2.07 (m, 1H).

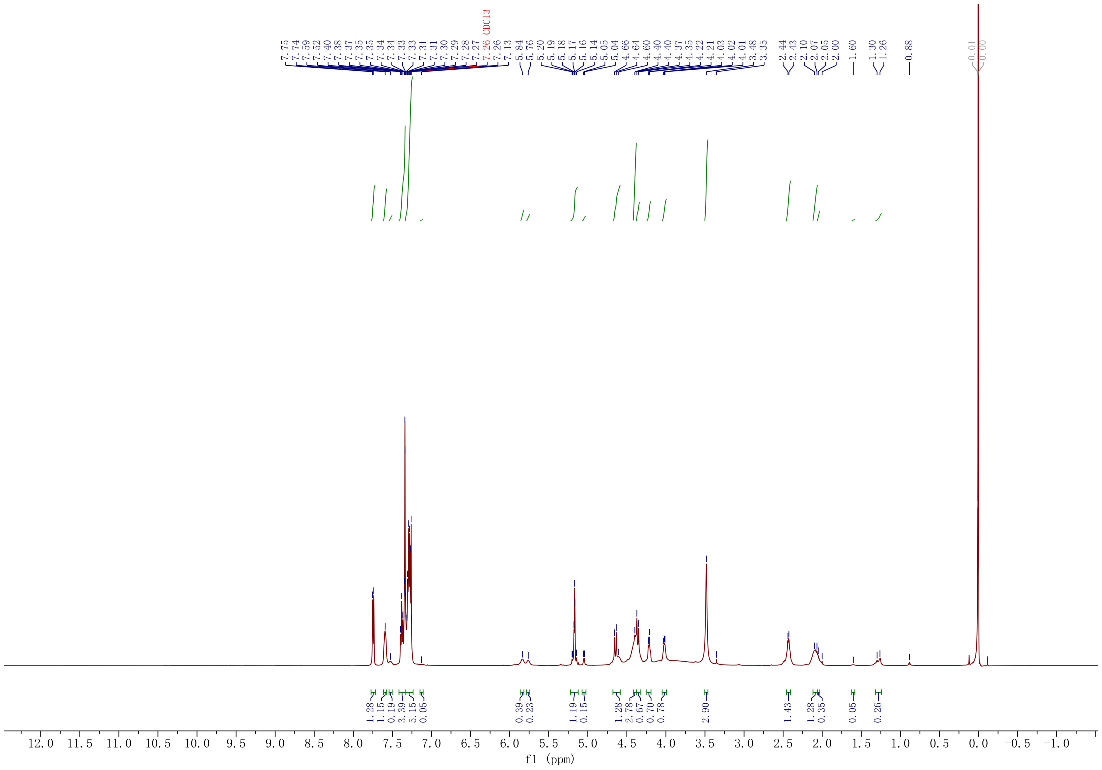

(6) Peptide standard synthesis

Compound S5 served as the protected amino acid for peptide synthesis. Peptide standards containing the 2HG modification were synthesized using a standard Fmoc-based solid-phase peptide synthesis workflow[7]. Note: Because HPLC showed only slight separation of the S4 isomers (similar hydrophobicity), isolating individual isomers caused substantial yield loss and insufficient material for peptide incorporation; therefore, S4 and its downstream product were used as an isomeric mixture.

References

1. Li, P. et al. *Analytical Chemistry* **93 (9)**, 4342-4350 (2021).

2. Jiang, Y. et al. *Nature* **567**, 257-261 (2019).

3. Abramson, J. et al. *Nature* **630**, 493–500 (2024).

4. Liang, B.W. et al. *Research* **6,** 0078 (2023).

5. Barber, K.W. & Rinehart, J. *Nature Chemical Biology* **14**, 188-192 (2018).

6. Li, G., Panday, S.K. & Alexov, E. *International Journal of Molecular Sciences* **22 (2)**, 606 (2021).

7. Hostetler, M.A. et al. *ACS Chemical Biology* **16 (11)**, 2604–2611 (2021).
