# Supplementary material for: Discovery of chirally dependent protein modifications by D- and L-2-hydroxyglutarates
Source: Nat Chem. 2026 Mar 17;18(6):1033–41. doi: 10.1038/s41557-026-02093-x (PMC13193722; doi:10.1038/s41557-026-02093-x)
Supplement: Supplementary file 2 — Reporting Summary [file 41557_2026_2093_MOESM2_ESM.pdf]

nature portfolio

Corresponding author(s): W. Andy Tao

Last updated by author(s): Jan 1, 2025

Reporting Summary

Nature Portfolio wishes to improve the reproducibility of the work that we publish. This form provides structure for consistency and transparency in reporting. For further information on Nature Portfolio policies, see our Editorial Policies and the Editorial Policy Checklist.

Statistics

For all statistical analyses, confirm that the following items are present in the figure legend, table legend, main text, or Methods section.

n/a	Confirmed	
☐	☒	The exact sample size (*n*) for each experimental group/condition, given as a discrete number and unit of measurement
☐	☒	A statement on whether measurements were taken from distinct samples or whether the same sample was measured repeatedly
☐	☒	The statistical test(s) used AND whether they are one- or two-sided<br>*Only common tests should be described solely by name; describe more complex techniques in the Methods section.*
☒	☐	A description of all covariates tested
☒	☐	A description of any assumptions or corrections, such as tests of normality and adjustment for multiple comparisons
☐	☒	A full description of the statistical parameters including central tendency (e.g. means) or other basic estimates (e.g. regression coefficient) AND variation (e.g. standard deviation) or associated estimates of uncertainty (e.g. confidence intervals)
☐	☒	For null hypothesis testing, the test statistic (e.g. *F*, *t*, *r*) with confidence intervals, effect sizes, degrees of freedom and *P* value noted<br>*Give P values as exact values whenever suitable.*
☒	☐	For Bayesian analysis, information on the choice of priors and Markov chain Monte Carlo settings
☒	☐	For hierarchical and complex designs, identification of the appropriate level for tests and full reporting of outcomes
☒	☐	Estimates of effect sizes (e.g. Cohen's *d*, Pearson's *r*), indicating how they were calculated

Our web collection on statistics for biologists contains articles on many of the points above.

Software and code

Policy information about availability of computer code

Data collection	MassHunter 10.1 (Agilent) and Bruker Compass Hystar 6.3 (Bruker) were used to collect the LC-MS data.
Data analysis	Bruker Compass DataAnalysis 6.1 were used for manually validation of peptide peaks; FragPipe software 21.0 were used for proteomic data analysis; Graphpad Prism 8.0 was used for statistical analysis throughout this study; Origin 2019b was used for plotting MS/MS spectra and chromatograms; Chemdraw 25.0.2 was used for drawing chemical structures; Protein structures were generated using AlphaFold 3 and crystal structures of proteins were illustrated using Pymol 2.1.1; illustrative workflows were made using BioRender.

For manuscripts utilizing custom algorithms or software that are central to the research but not yet described in published literature, software must be made available to editors and reviewers. We strongly encourage code deposition in a community repository (e.g. GitHub). See the Nature Portfolio guidelines for submitting code & software for further information.

Data

Policy information about availability of data

All manuscripts must include a data availability statement. This statement should provide the following information, where applicable:

- Accession codes, unique identifiers, or web links for publicly available datasets
- A description of any restrictions on data availability
- For clinical datasets or third party data, please ensure that the statement adheres to our policy

The raw mass spectrometry proteomics data, protein identification and quantification results have been deposited with the ProteomeXchange Consortium via the

PRIDE partner repository with the dataset identifier PXD072530. The Supplementary Tables are published alongside this paper. The reviewed human fasta database was downloaded from UniProt in March 2024 (https://www.uniprot.org/uniprotkb). Source data are provided with this paper.

Research involving human participants, their data, or biological material

Policy information about studies with human participants or human data. See also policy information about sex, gender (identity/presentation), and sexual orientation and race, ethnicity and racism.

Reporting on sex and gender	N/A
Reporting on race, ethnicity, or other socially relevant groupings	N/A
Population characteristics	N/A
Recruitment	N/A
Ethics oversight	N/A

Note that full information on the approval of the study protocol must also be provided in the manuscript.

Field-specific reporting

Please select the one below that is the best fit for your research. If you are not sure, read the appropriate sections before making your selection.

☒ Life sciences ☐ Behavioural & social sciences ☐ Ecological, evolutionary & environmental sciences

For a reference copy of the document with all sections, see nature.com/documents/nr-reporting-summary-flat.pdf

Life sciences study design

All studies must disclose on these points even when the disclosure is negative.

Sample size	No statistical methods were used to predetermine sample size. For 2HG-modified peptide identification and endogenous 2HG profiling experiments, sample sizes for cell samples were determined based on the minimal number of biologically independent replicates required to identify significant changes (n=3 biological replicates per group). As demonstrated in the extensive proof-of-concept applications, the sample size is sufficient to generate confident targets. To address the false targets of 2HG-modified peptide identification, we profiled D2HG / L2HG modified peptides across multiple D2HG- or L2HG-accumulating conditions and cross-validated candidate peptides between groups. High-confidence D- and L2HG-modified peptides were defined using a stepwise workflow (Supplementary Note 2).<br>For method development, sample sizes were two technical replicates for method optimization and five technical replicates for method stability assessment. For technical replicates, a single pooled starting sample was split into equal aliquots, and each aliquot underwent independent enrichment. Biological replicates were not used for method optimization because biological variability can obscure technical performance.
Data exclusions	No data was excluded from our analysis.
Replication	Conclusions were drawn from reproducible effects in all replicates of the data sets, Respective numbers of available replicates are indicated in the manuscript, methods, and corresponding figure legends.
Randomization	Randomization was not applicable because this study did not involve allocation of human participants, animals, or other live organisms. Treatments were applied to parallel cell-culture samples prepared and handled under standardized conditions, without meaningful random assignment. Samples were processed in parallel to minimize batch and plate-position effects.
Blinding	Blinding was not relevant here because group allocation was not involved in this study.

Reporting for specific materials, systems and methods

We require information from authors about some types of materials, experimental systems and methods used in many studies. Here, indicate whether each material, system or method listed is relevant to your study. If you are not sure if a list item applies to your research, read the appropriate section before selecting a response.

Materials & experimental systems

n/a	Involved in the study
☒	☐ Antibodies
☐	☒ Eukaryotic cell lines
☒	☐ Palaeontology and archaeology
☒	☐ Animals and other organisms
☒	☐ Clinical data
☒	☐ Dual use research of concern
☒	☐ Plants

Methods

n/a	Involved in the study
☒	☐ ChIP-seq
☒	☐ Flow cytometry
☒	☐ MRI-based neuroimaging

Eukaryotic cell lines

Policy information about cell lines and Sex and Gender in Research

Cell line source(s)	H293T (Cat. CRL-3216) were obtained from American Type Culture Collection (ATCC). ICC cell lines with different genotypes (wild type SSP25 cells and IDH1 mutant RBE cells) were generously provided by Dr. Nabeel Bardeesy (Massachusetts General Hospital, Harvard Medical School, Boston, Massachusetts).
Authentication	H293T cells (ATCC CRL-3216) were authenticated by the supplier by STR profiling. SSP25 and RBE were authenticated by Dr. Nabeel Bardeesy's laboratory by STR profiling.
Mycoplasma contamination	All cells were tested negative for mycoplasma.
Commonly misidentified lines (See ICLAC register)	No commonly misidentified cell lines were used in this study.

Plants

Seed stocks	N/A
Novel plant genotypes	N/A
Authentication	N/A
